# Segmentation of Shallow Slow Slip Events at the Hikurangi Subduction Zone Explained by Along‐Strike Changes in Fault Geometry and Plate Convergence Rates

**DOI:** 10.1029/2021JB022913

**Published:** 2022-01-21

**Authors:** Andrea Perez‐Silva, Yoshihiro Kaneko, Martha Savage, Laura Wallace, Duo Li, Charles Williams

**Affiliations:** ^1^ School of Geography, Environment and Earth Sciences Victoria University of Wellington Wellington New Zealand; ^2^ Department of Geophysics Kyoto University Kyoto Japan; ^3^ GNS Science Lower Hutt New Zealand; ^4^ Institute for Geophysics University of Texas at Austin Austin TX USA; ^5^ Department of Earth and Environmental Sciences Ludwig‐Maximilians‐Universität München München Germany

**Keywords:** slow slip events, subduction zone, numerical simulations, Hikurangi margin, rate‐and‐state friction, segmentation of slow slip events

## Abstract

Over the last two decades, geodetic and seismic observations have revealed a spectrum of slow earthquakes along the Hikurangi subduction zone in New Zealand. Of those, shallow slow slip events (SSEs) that occur at depths of less than 15 km along the plate interface show a strong along‐strike segmentation in their recurrence intervals, which vary from ∼1 yr from offshore Tolaga Bay in the northeast to ∼5 yr offshore Cape Turnagain ∼300 km to the southwest. To understand the factors that control this segmentation, we conduct numerical simulations of SSEs incorporating laboratory‐derived rate‐and‐state friction laws with both planar and non‐planar fault geometries. We find that a relatively simple model assuming a realistic non‐planar fault geometry reproduces the characteristics of shallow SSEs as constrained by geodetic observations. Our preferred model captures the magnitudes and durations of SSEs, as well as the northward decrease of their recurrence intervals. Our results indicate that the segmentation of SSE recurrence intervals is favored by along‐strike changes in both the plate convergence rate and the downdip width of the SSE source region. Modeled SSEs with longer recurrence intervals concentrate in the southern part of the fault (offshore Cape Turnagain), where the plate convergence rate is lowest and the source region of SSEs is widest due to the shallower slab dip angle. Notably, the observed segmentation of shallow SSEs cannot be reproduced with a simple planar fault model, which indicates that a realistic plate interface is an important factor to account for in modeling SSEs.

## Key Points

- We simulate shallow slow slip events (SSEs) at Hikurangi margin using a rate‐and‐state fault model that incorporates three‐dimensional non‐planar geometry
- Model reproduces the magnitudes, duration, and slip of shallow SSEs, as well as the along‐strike change in their recurrence intervals
- Along‐strike changes in the fault geometry and the plate convergence rate may control the segmentation of shallow SSEs

## 1 Introduction

Slow slip events (SSEs) are transient episodes of aseismic slip with longer durations and slower slip velocities than typical earthquakes. An SSE can generate millimeters to tens of centimeters of slip on a fault over periods of days to years (Schwartz & Rokosky, 2007). These events often occur at quasi‐periodic intervals, spanning months to several years (Beroza & Ide, 2011), and play a significant role in the earthquake cycle where they occur, as they release part of the accumulated strain energy (e.g., Araki et al., 2017; Bartlow, 2020; Radiguet et al., 2012), and may influence the timing of earthquake occurrence (Kaneko et al., 2018; Kato et al., 2012; Obara & Kato, 2016; Ruiz et al., 2014). SSEs have been detected in various tectonic settings including strike‐slip faults (e.g., Chamberlain et al., 2014; Linde et al., 1996; Rousset et al., 2019; Wech et al., 2012; Wei et al., 2013) and volcanic islands (Brooks et al., 2006; Cervelli et al., 2002; Segall et al., 2006), however, they are most commonly observed in subduction zones (e.g., Dixon et al., 2014; Dragert et al., 2004; Fu & Freymueller, 2013; Hirose & Obara, 2005; Lowry et al., 2001; Obara et al., 2004; Ohta et al., 2006; Outerbridge et al., 2010; Radiguet et al., 2016; Wallace & Beavan, 2010; Wei et al., 2012).

**Figure 1:**
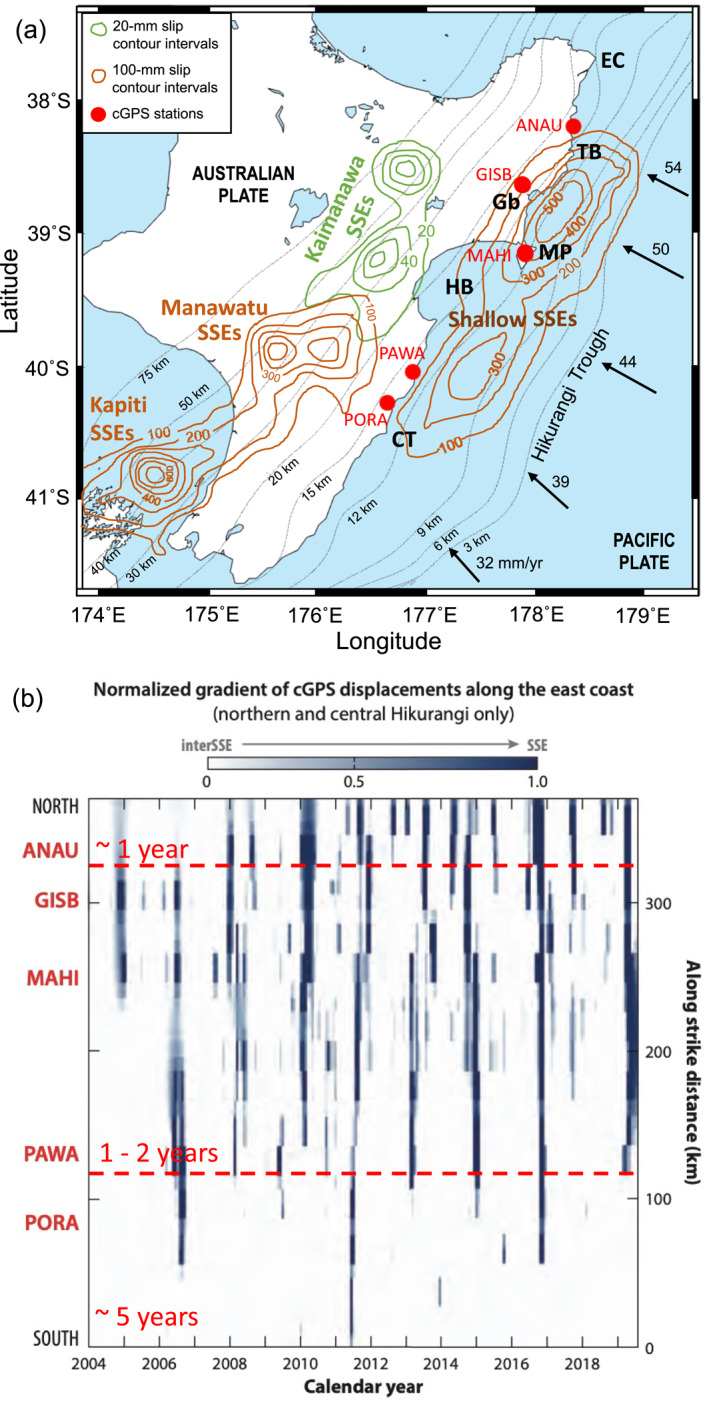
(a) Cumulative slow slip in the North Island of New Zealand for the 2002–2014 period (contours from Figure 1 in Wallace, 2020). Brown contours are 100‐mm slip contour intervals. Green contours show 20‐mm slip intervals. Red dots show the location of continuous GPS (cGPS) stations ANAU, GISB, MAHI, PAWA, and PORA, labeled in (b). Black arrows indicate the plate convergence rate in mm/yr (data from Wallace, Beavan, Bannister, & Williams, 2012). Thin black lines are the depth contour (below sea level) of the subducting plate interface (based on Williams et al., 2013). Abbreviations: EC, East Cape; TB, Tolaga Bay; Gb, Gisborne; MP, Mahia Peninsula; HB, Hawke’s Bay; CT, Cape Turnagain. (b) Change in rate of motion of GeoNet cGPS stations as a normalized gradient. Darker colors represent fastest rate change, indicative of SSEs. White color indicate inter SSE period. The time series are projected along‐strike (*y*‐axis). Red labels on *y*‐axis indicate the location of the cGPS stations shown in (a). Dashed red lines divide the along‐strike distance into three segments based on the change in the recurrence interval of SSEs. The estimated recurrence interval at each segment is shown in red. Figure modified from Wallace (2020).

The Hikurangi subduction zone, where the Pacific Plate subducts beneath the Australian plate (Figure 1a), is exceptional in the diversity of SSE characteristics that occur there (Wallace & Beavan, 2010). At Hikurangi, SSEs have been detected at both shallow and deep depths along the plate interface (Douglas et al., 2005; Wallace, 2020; Wallace & Beavan, 2010; Wallace, Beavan, Bannister, & Williams, 2012). Deep SSEs (<50 km depth) have been observed in the Kapiti and Manawatu regions, as well as beneath the Kaimanawa ranges (Figure 1a); whereas shallow SSEs (<15 km depth) concentrate offshore the east coast of the North Island, from East Cape to Cape Turnagain (Figure 1a). Deep and shallow SSEs at Hikurangi exhibit contrasting source properties. Deep Hikurangi SSEs typically last 1–2 yr, reach magnitudes larger than ∼*M*_w_7.0 and recur every ∼5 yr (Bartlow et al., 2014; Ikari et al., 2020; Wallace & Beavan, 2010; Wallace, Beavan, Bannister, & Williams, 2012). In contrast, shallow Hikurangi SSEs have shorter durations (1–4 weeks), lower magnitudes (*M*_w_6.0–6.6; Ikari et al., 2020) and their recurrence intervals range from ∼1 to 5 yr (Douglas et al., 2005; Wallace & Beavan, 2010; Wallace, Beavan, Bannister, & Williams, 2012). For both deep and shallow Hikurangi SSEs, slip on the plate boundary can be centimeters to tens of centimeters (Ikari et al., 2020; Wallace & Beavan, 2010).

Shallow SSEs along the Hikurangi margin display a marked along‐strike segmentation in their recurrence intervals, which increase southward along the strike of the margin (Wallace, 2020; Wallace & Beavan, 2010; Wallace, Beavan, Bannister, & Williams, 2012). In the northern part of the margin, offshore Tolaga Bay, SSEs recur most frequently, with one to two events detected each year. In the central part, offshore Gisborne and Hawke’s Bay regions, the recurrence time is ∼1–2 yr, whereas in the south, offshore Porangahau and Cape Turnagain, they occur every ∼5 yr. Figure 1b illustrates the change in the recurrence interval of shallow Hikurangi SSEs along the margin. We note that along‐strike segmentation of deep SSEs at Hikurangi is not as well constrained as for shallow SSEs. Notably, along‐strike changes in the recurrence interval of SSEs have been reported in other subduction zones, such as Alaska (Fu et al., 2015; Li et al., 2018; Wei et al., 2012), Cascadia (Brudzinski & Allen, 2007), Nankai (Obara, 2010; Takagi et al., 2019), and Mexico (Graham et al., 2016).

Several factors have been proposed to explain SSE segmentation based on numerical modeling, and geodetic and seismic observations. Numerical simulations indicate that along‐strike changes in effective normal stress (the difference between lithostatic load and pore fluid pressure) could lead to segmentation of SSE recurrence intervals, with shorter intervals associated with lower effective normal stress (Li et al., 2018; Liu, 2014). Another model suggests that changes in the plate convergence rate and the width of the source region of SSEs may also affect the periodicity of these events (Shibazaki et al., 2012). Simulations of SSE cycles assuming a realistic plate geometry showed links between spatial variations in the plate dip and strike angles, and the segmentation of SSEs (Li & Liu, 2016). In addition, geodetic observations found correlations between the location of locked asperities in the updip area and the segmentation of SSE recurrence interval and cumulative slip (Takagi et al., 2019). Other potential causes of SSE segmentation include spatial variations in pore fluid pressure due to silica enrichment (Audet & Bürgmann, 2014), and along‐strike changes in the density of geological terranes in the overriding plate (Brudzinski & Allen, 2007; Li & Liu, 2017). A review of the factors that may affect the segmentation of SSEs can be found in Li et al. (2018). Yet it is still uncertain which of these factors control the segmentation of shallow SSEs along the Hikurangi margin.

In this study, we conduct numerical simulations to understand the factors that control the segmentation of shallow SSEs in Hikurangi. Our modeling approach accounts for continuum elasticity and a realistic three‐dimensional (3D) geometry of the plate interface, where the fault resistance to sliding is described by laboratory‐derived rate‐and‐state friction laws. We note that while Shibazaki et al. (2019) modeled both shallow and deep Hikurangi SSEs and focused on their interactions with large earthquakes using a similar approach, the cause of the segmentation of shallow SSEs was not investigated. This article is structured as follows. Section 2 introduces the method and parameter setup of our numerical model. In Section 3, we conduct an exploration of the controlling model parameters and describe a preferred model that reproduces the observed characteristics of shallow Hikurangi SSEs. In Section 4, we discuss the factors that control the along‐strike segmentation of shallow SSEs and the implications for other relevant observations.

## 2 Model Setup

To simulate SSE cycles, we use the numerical code developed by Li and Liu (2016). There are three main ingredients of this modeling approach: (a) it implements a quasi‐dynamic formulation of traction and slip as defined by Rice (1993), (b) the fault constitutive response is given by rate‐and‐state friction laws with the aging form of state‐variable evolution (Dieterich, 1979), and (c) it enables the incorporation of a 3D non‐planar fault geometry. The simulation code implements the Boundary Integral Element Method and accounts for Earth’s free surface. We describe the governing equations of the model in the Text S1 in Supporting Information S1.

In the rate‐and‐state friction formulation, the evolution of the steady‐state friction coefficient in response to a step change in slip velocity depends on the lab‐derived friction parameters *a* and *b* (Blanpied et al., 1998; Dieterich, 1979). Materials with *a* − *b* > 0 are velocity‐strengthening (VS), such that an increase in slip velocity results in an increase in steady‐state friction, thus stabilizing slip. Materials with *a* − *b* < 0 are velocity‐weakening (VW); increasing the slip velocity causes a decrease in steady‐state friction, and slip can be unstable (seismic) or conditionally stable (Scholz, 1998).

The slip behavior of the fault largely depends on the effective fault stiffness ratio *W*/h* (Barbot, 2019; Liu & Rice, 2007), where *W* is the downdip width of the VW region under low effective normal stress (Section 2.2) and h* is the critical patch size to generate unstable slip [Equation (4) in Text S1 in Supporting Information S1, Rubin & Ampuero, 2005]. This fault stiffness ratio depends on the Poisson’s ratio (*ν*), shear modulus (*μ*), effective normal stress $\left({\bar{\sigma }}_{n}\right)$, rate‐and‐state parameters (*d*_c_ and *a* − *b*), and fault geometry. For *W*/h* ≫ 1 unstable slip may occur, while much smaller values point to stable slip (Liu & Rice, 2007). Previous numerical models (Li & Liu, 2016; Liu & Rice, 2007, 2009) have found that a *W*/h* close to unity favors episodic slow slip behavior. In addition to *W*/h*, the ratio *a*/*b* has also been shown to control the fault slip behavior (Barbot, 2019; Rubin, 2008).

### 2.1 Fault Geometry

The model assumes a 3D fault geometry of the Hikurangi plate interface based on Williams et al. (2013), which was constrained by earthquake locations and seismic reflection images. Our model fault extends 500 km along the Hikurangi margin (latitudes 41°–37°S) and covers the depth range from the trench, at 2.5 km depth below sea level, down to 30 km (Figure S1 in Supporting Information S1). We discretize the slab surface by 84,906 triangular elements, each with an area of ∼1 km^2^, using Cubit/Trelis Software (https://www.coreform.com/). Given that the smallest value of the critical nucleation size (h*) is 80 km, h*/*dx* ∼80, where *dx* is the average length of the triangle edges (1 km). Such discretization ensures that h* is well resolved, and is similar to that used in previous 3D simulations of SSEs (Li et al., 2018; Li & Liu, 2016, 2017; Perez‐Silva et al., 2021). We note that our model neglects normal stress changes induced by slip on the non‐planar fault, similar to previous SSE models in non‐planar faults (e.g., Li & Liu, 2016; Shibazaki et al., 2012, 2019). This assumption is not expected to significantly affect the model results, as we consider a smooth plate‐boundary fault and normal stress changes induced by SSEs are relatively small.

### 2.2 Model Parameters

To account for the shallow depths of SSEs, we set the shear modulus to 15 GPa, which is slightly above but comparable to the recently inferred range (6–14 GPa) at central Hikurangi using full waveform inversion of controlled‐source seismic data (Arnulf et al., 2021). We assume a Poisson’s ratio of 0.25, corresponding to a Poisson solid.

**Figure 2:**
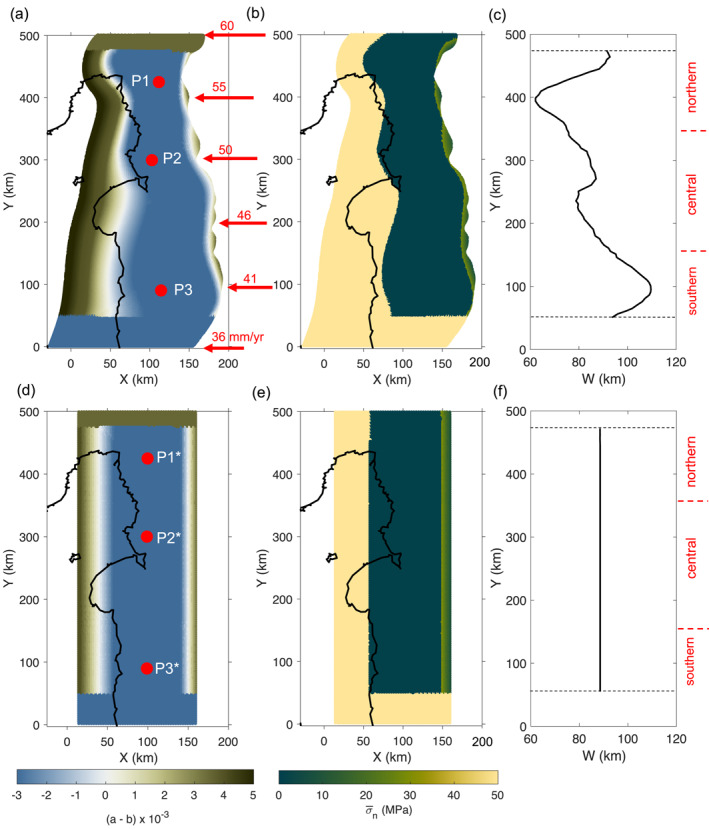
Model setup of (a)–(c) non‐planar and (d)–(f) planar geometries with the map view distribution of (a and d) friction parameter (*a *– *b*) and (b and e) ${\bar{\sigma }}_{n}$ on the fault. Note that while the model with (*a *– *b*) = −0.003 and ${\bar{\sigma }}_{n}$ = 1 MPa in the SSE zone is shown in this figure, we also consider the case with (*a *– *b*) = −0.000 3 and ${\bar{\sigma }}_{n}$ = 10 MPa (Figure S2 in Supporting Information S1). Red arrows in (a) indicate the plate convergence rate along‐strike in mm/yr. Along‐strike variation of *W* for (c) non‐planar and (f) planar geometry. Dashed black lines in (c and f) mark the along‐strike limit of the SSE zone. Red labels on the right of (c and f) indicate three segments into which the fault geometry is divided: northern (350 km < Y< 475 km), central (150 km <Y < 350 km), and southern (50 km < Y < 150 km). Red circles in (a and d) show reference points P1–P3 and P1*–P3*.

The fault is loaded by spatially non‐uniform plate motion. We set the plate convergence rate perpendicular to the trench and increasing linearly northward from 36 to 60 mm/yr along the strike of the fault (red arrows in Figure 2a; see also Figure S1 in Supporting Information S1), which is consistent with the estimation from modeling of the campaign GPS velocity field (Wallace, Barnes, et al., 2012; Wallace et al., 2004). Slip partitioning occurs at the Hikurangi subduction margin, whereby the margin‐parallel component of plate motion is accommodated by strike‐slip faulting and tectonic‐block rotation of the eastern North Island, and the shallow plate interface is dominated by trench‐normal convergence (Wallace et al., 2004, 2009).

We define the distribution of the friction parameter (*a* − *b*) along the fault dip such that the VW region roughly matches with the along‐dip extent of observed shallow SSEs (Figure 1a). Figure 2a and Figure S2a in Supporting Information S1 show the map view of the (*a* − *b*) distribution. We set a uniform value of (*a* − *b*)_vw_ = −0.003 or −0.0003 from 4 km depth until the downdip limit of the slip contours of shallow SSEs. The assumed (*a* − *b*)_vw_ are comparable to those obtained from friction experiments on incoming sediments to Hikurangi margin, where it ranges from −0.000 4 to −0.001 5 (Rabinowitz et al., 2018) and from −0.001 9 to −0.003 (Ikari et al., 2020). Outside the source region of SSEs, both updip and downdip, we assume a linear increase of (*a* − *b*) from VW (*a* − *b* < 0) to VS (*a* − *b* > 0) conditions (Figure 2a).

Low effective normal stress, or equivalently, high pore fluid pressures, have been adopted by several numerical models to reproduce SSE properties (e.g., Li et al., 2018; Li & Liu, 2016, 2017; Liu, 2014; Liu & Rice, 2007, 2009; Matsuzawa et al., 2010, 2013; Shibazaki et al., 2012, 2019; Shibazaki & Shimamoto, 2007; Wei et al., 2018). This assumption is based on inferred high pore pressure conditions in the source regions of SSEs (Audet et al., 2009; Audet & Kim, 2016; Kodaira et al., 2004; Song et al., 2009), and it is also supported by geophysical observations at Hikurangi (Bassett et al., 2014; Eberhart‐Phillips & Bannister, 2015; Eberhart‐Phillips et al., 2017; Heise et al., 2013, 2017). Following this assumption, the effective normal stress $\left({\bar{\sigma }}_{n}\right)$ is set to a low value (${\bar{\sigma }}_{n}$ = 1 or 10 MPa) in the source region of SSEs (Figure 2b and Figure S2b in Supporting Information S1). For simplicity, we do not assume along‐dip changes in ${\bar{\sigma }}_{n}$ within this region.

We refer to the region with low ${\bar{\sigma }}_{n}$ and VW conditions as the *SSE zone*. Farther downdip of the SSE zone, ${\bar{\sigma }}_{n}$ = 50 MPa (Figure 2b and Figure S2b in Supporting Information S1). From the trench (at 2.5 km depth) down to 4 km depth, ${\bar{\sigma }}_{n}$ increases from 7 to 30 MPa (Figure 2b and Figure S2b in Supporting Information S1). Since the updip extent of SSEs is not well constrained by observations, this assumption is set to avoid SSEs propagating all the way to the trench. To test the viability of this assumption, we additionally consider a case with low ${\bar{\sigma }}_{n}$ starting from the trench (Section 3.2.5).

The characteristic slip distance *d*_c_ is set to scale with ${\bar{\sigma }}_{n}$ and (*a* − *b*) on the fault [Equation (4) in Text S1 in Supporting Information S1], while h* is assumed constant. Within the SSE zone, *d*_c_ is constant as *a*, *b*, and ${\bar{\sigma }}_{n}$ are uniform in this region. Outside the SSE zone, *d*_c_ increases with depth until it reaches 2 m, after which it remains constant. The increase of *d*_c_ with depth outside the SSE zone is motivated by computational efficiency and produces the same results for shallow SSEs as using a constant *d*_c_ for all depths (Lapusta et al., 2000).

As the Hikurangi plate interface is estimated to be strongly coupled in the southern part of the margin (Wallace & Beavan, 2010), we set VW conditions (*a* − *b* < 0) and ${\bar{\sigma }}_{n}$ = 50 MPa in that region (0 km < *Y* < 50 km; Figures 2a and 2b, respectively. See also Figure S2 in Supporting Information S1). This parameter setting is not equivalent to a kinematic, fully locked condition, as it allows slip to penetrate into the locked zone. The plate coupling in the northern part of the margin, further north from East Cape, is not well constrained; here we assume VS conditions in that region (*a* − *b* > 0 for 475 km < *Y* < 500 km in Figure 2a and Figure S2a in Supporting Information S1), which would lead to stable sliding. We also examine alternative parameterizations for the northern and southern ends of the model fault and discuss the results in Section 3.2.5.

The model parameter *W*, which measures the downdip width of the SSE zone, varies along the strike of the fault as shown in Figure 2c. Being an along‐dip distance, this parameter is inversely proportional to the dip angle of the plate interface. Notably, *W* is widest in the southern part of the margin (Figure 2c), consistent with a shallower dip angle of the plate‐boundary fault in that region (Barker et al., 2009). We note that as h* is assumed uniform along strike, a change in *W* leads to a change in the effective fault stiffness ratio *W*/h*.

## 3 Model Results

### 3.1 Parameter Exploration

We first perform a total of 63 simulations, each of which takes at least 24 hr on 53 physical cores of the New Zealand eScience Infrastructure’s Cray XC50 supercomputer, to explore a wide range of model parameters and identify a set of models that result in SSEs along the entire margin. As expected, depending on friction parameters *a*/*b* and the ratio *W*/h*, the model leads to three different slip behaviors: (a) stable creep, (b) SSEs (*V* > ∼3 Vpl_ref_ or 0.39 mm/day), where Vpl_ref_ = 45 mm/yr is a reference plate convergence velocity, and (c) seismic events (*V* > 5 mm/s). The slip behavior is classified as “seismic” when at least one seismic event arises in the first 20 SSE cycles, that is, if the maximum velocity on the fault exceeds 5 mm/s before the first 20 SSEs have emerged. This condition is set to distinguish this slip pattern from simulations where SSEs are the primary mode of slip, noting that seismic events also arise after many (≫20) SSE cycles in a few simulations classified as SSEs. Given that simulating earthquakes is computationally demanding with the numerical approach used in this study, we do not analyze SSE cycles after the emergence of seismic events.

**Figure 3:**
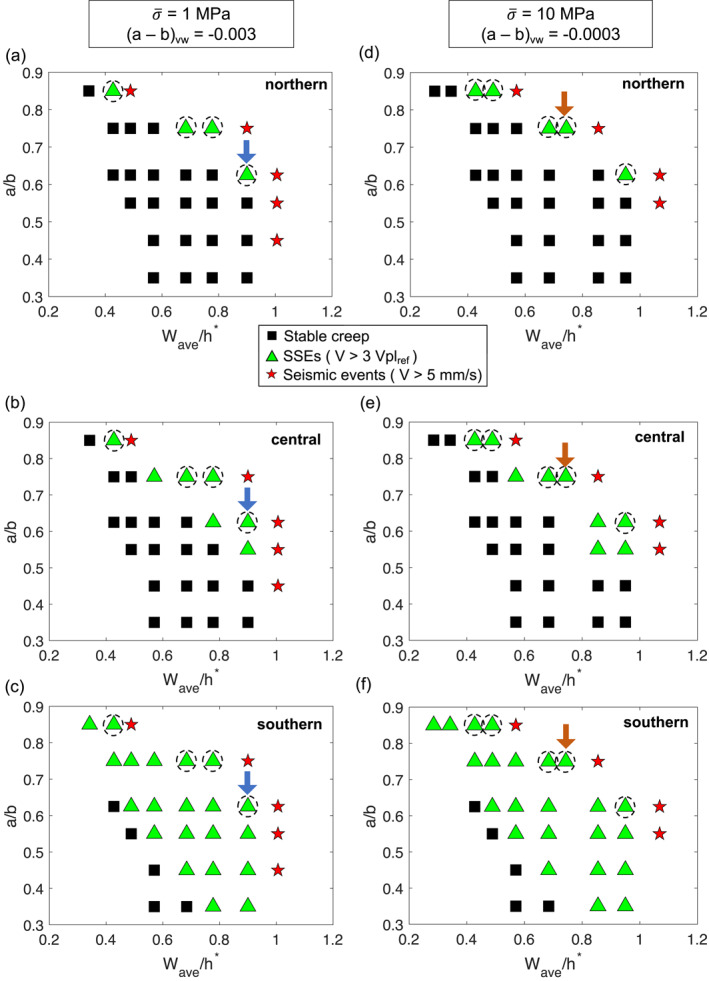
Simulated slip patterns (stable creep, SSEs, or seismic events) for different combinations of *a*/*b* and *W*_ave_/h* parameters. *W*_ave_ = 87.5 km is the average *W* along‐strike from Figure 2c, and we explore h* in the range of 80–300 km. For simplicity we assume *W*/h* = *W*_ave_/h*, but this ratio varies along the strike of the margin in proportion to Figure 2c. Northern (a and d), central (b and e), and southern (c and f) correspond to the segments defined along the strike of the fault (red labels on the right of Figure 2c). (a)–(c) Simulation cases with ${\bar{\sigma }}_{n}$ = 1 MPa and (*a* − *b*)_vw_ = −0.003, and (d)–(f) with ${\bar{\sigma }}_{n}$ = 10 MPa and (*a* − *b*)_vw_ = −0.000 3. Blue arrow indicates the preferred model. Orange arrow points to the preferred model for ${\bar{\sigma }}_{n}$ = 10 MPa in the SSE zone. Dashed circles highlight simulation cases where SSEs emerge in all three segments. All simulations were carried out assuming the *W* distribution along‐strike shown in Figure 2c.

Phase diagrams in Figures 3a–3f show the slip behavior with respect to *a*/*b* and *W*_ave_/h*, where *W*_ave_ = 87.5 km is the average *W* along‐strike, calculated from the profile in Figure 2c. We explore h* within the range of 80–300 km, corresponding to ∼0.3 $<\;W_{\text{ave}}/h^{*}\;<\;∼$1.1. We present the results for two different values of ${\bar{\sigma }}_{n}$ and (*a* − *b*)_vw_ in the SSE zone (textbox on top of Figure 3), noting that for both models, the products $a{\bar{\sigma }}_{n}$ and $b{\bar{\sigma }}_{n}$ are the same. As the slip behavior often varies along the strike of the fault, to describe these variations we divide the fault into three major segments: northern, central, and southern (red labels on the right in Figure 2c). These segments loosely correspond to the along‐strike ranges of SSE recurrence intervals estimated from observations (dashed red lines in Figure 1b). For each simulation in Figure 3, we show the slip behavior at each of these segments in separate plots (e.g., Figures 3a–3c), noting that they correspond to the same simulation case.

Phase diagrams for ${\bar{\sigma }}_{n}$ = 1 MPa and (*a* − *b*)_vw_ = −0.003 (Figures 3a–3c) are qualitatively similar to those with ${\bar{\sigma }}_{n}$ = 10 MPa and (*a* − *b*)_vw_ = −0.000 3 (Figures 3d–3f), as expected from the rate‐and‐state friction laws [Equations (2) and (3) in Text S1 in Supporting Information S1]. The minor differences between them could be due to the location of the down‐dip limit of the VW‐VS transition, which is slightly shallower in the model with ${\bar{\sigma }}_{n}$ = 10 MPa and (*a* − *b*)_vw_ = −0.000 3 (Figure S2a in Supporting Information S1). Note that this difference does not affect the distribution of *W*, which is the same in both model setups (Figure 2c). Regarding the fault slip behavior, in both cases the slip pattern changes along the strike of the fault. Notably, in the northern segment most simulation cases exhibit stable creep (black squares in Figures 3a and 3d), whereas in the southern segment SSEs are the predominant slip pattern (green triangles in Figures 3c and 3f). Figure S3 in Supporting Information S1 shows an example of the along‐strike variation in the slip pattern.

SSE slip behavior emerges in all three segments only in a few simulations, which are indicated by the black dashed circles in Figure 3. Among these cases, the characteristics of SSEs vary; we select the preferred model as the one that produces SSEs of cumulative slip, duration, magnitude, and recurrence interval similar to those observed in the shallow Hikurangi margin, that is, centimeters to tens of centimeters, 1–4 weeks, ∼*M*_w_6.0–6.6 and increasing from ∼1 to ∼5 yr southward along the margin, respectively. The parameters of the preferred model (blue arrow in Figure 3) are given in Table 1. In the following, we describe the characteristics of SSEs in the preferred model and compare them with observations.

**Table 1:** List of Parameters for Preferred Model

Definition	Parameters	Values
Nucleation size	h*	95 km (115 km)^a^
Characteristic slip distance	*d*_c_	8.39 mm (6.77 mm)
Effective normal stress in the SSE zone	${\bar{\sigma }}_{n}$	1 MPa (10 MPa)
Friction parameter	(*a* − *b* *)* *_vw_*	−0.003 (−0.000 3)
Direct effect	*a*	0.005 (0.000 9)
Shear modulus	*μ*	15 GPa
Poisson’s ratio	*ν*	0.25
Steady state friction coefficient at *V*_o_	*f*_o_	0.6

^a^ Values in parentheses correspond to the preferred model with ${\bar{\sigma }}_{n}$ = 10 MPa in the SSE zone only.

### 3.2 Characteristics of SSEs in the Preferred Model and Comparison With Observations

#### 3.2.1 Slip Velocity Evolution Along the Hikurangi Margin

**Figure 4:**
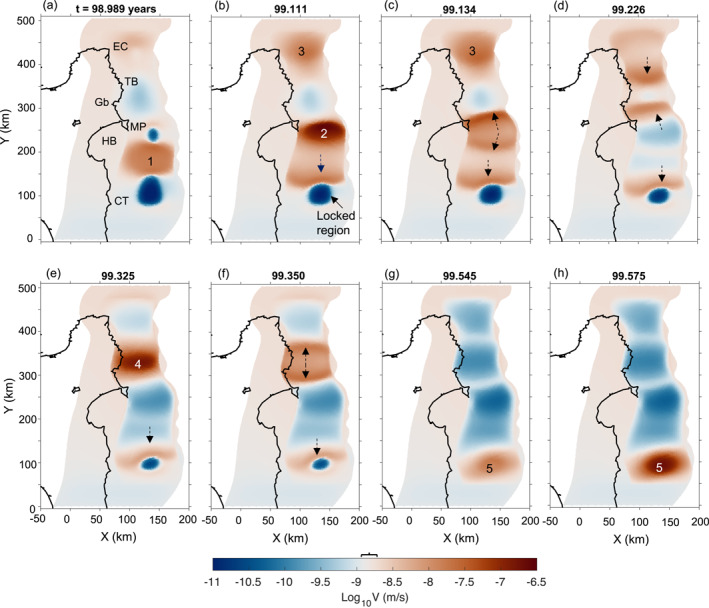
Snapshots of the slip velocity on the fault at eight successive time steps (see also Movie S1). Bold number on top of each figure indicates the simulation time in years. The lightest brown colors indicate regions that slide close to the plate convergence rate; dark blue corresponds to locked portions of the fault, that slip at one to two orders of magnitude below the plate rate, and brown to dark brown colors are indicative of SSEs, which emerge spontaneously as patches of high velocities. SSEs are numbered from 1 to 5 in order of their occurrence. Dashed arrows indicate migration of SSEs. Square bracket on top of colorbar indicates the range of plate convergence rates along *Y*. Abbreviations indicate reference locations: EC, East Cape; TB, Tolaga Bay; Gb, Gisborne; MP, Mahia Peninsula; HB, Hawke's Bay and CT, Cape Turnagain.

The maximum slip rate on the fault, *V*_max_, exhibits a complex evolution over time with peak velocities that span three orders of magnitude (from 10^−8.2^ to 10^−4.8^ m/s) and recur at variable periods (Figure S4a in Supporting Information S1). Such irregularity of *V*_max_ is due to changes in the slip rate along the fault. To visualize the slip behavior on the fault, we show snapshots of the slip velocity at successive time steps (Figure 4, Movie S1). As expected from the distribution of frictional properties on the fault (Figure 2a), the region with VS conditions slips steadily with velocities comparable to the plate rate (lightest brown color in Figure 4). The VW region with low ${\bar{\sigma }}_{n}$, on the other hand, slips periodically through SSEs, which emerge spontaneously as patches of higher velocities (brown to dark brown colors in Figure 4). The region in‐between SSEs is typically locked (dark blue colors in Figure 4). The degree of locking varies along the fault strike with the southern part of the margin being often more strongly locked. The southern portion of the fault (0 km < *Y* < 50 km) slides at a rate only slightly below the plate rate (lightest blue color between 0 and 50 km along‐strike in Figure 4).

The distribution of modeled SSEs is consistent with geodetic observations, in that SSEs emerge as patches of high slip velocity at different locations along the margin. In Figure 4, SSEs (labeled with numbers) emerge offshore Hawke’s Bay (SSE 1 in Figure 4a), East Cape and Mahia Peninsula (SSE 2 and 3 in Figure 4b), Gisborne (SSE 4 in Figure 4e), and Cape Turnagain (SSE 5 in Figures 4g and 4h). These SSEs migrate along the fault as slip fronts (dashed arrows in Figure 4) and interact with each other. For instance, two slip fronts migrate toward each other in the northern part of the fault (converging dashed black arrows in Figure 4d) and coalesce in a velocity peak (*V*_max_ > 10^−7^ m/s) that generates SSE 4 (Figure 4e). A slip front in Figure 4b slowly migrates southwards (dashed black arrow in Figures 4b–4f) and eventually leads to SSE 5 (Figure 4g). Geodetic observations have also identified along‐strike migration of SSEs, for example, during the 2011 East Coast sequence, SSEs migrated episodically ∼300 km along‐strike from offshore Castle Point to north of Gisborne (Wallace, Beavan, Bannister, & Williams, 2012).

**Figure 5:**
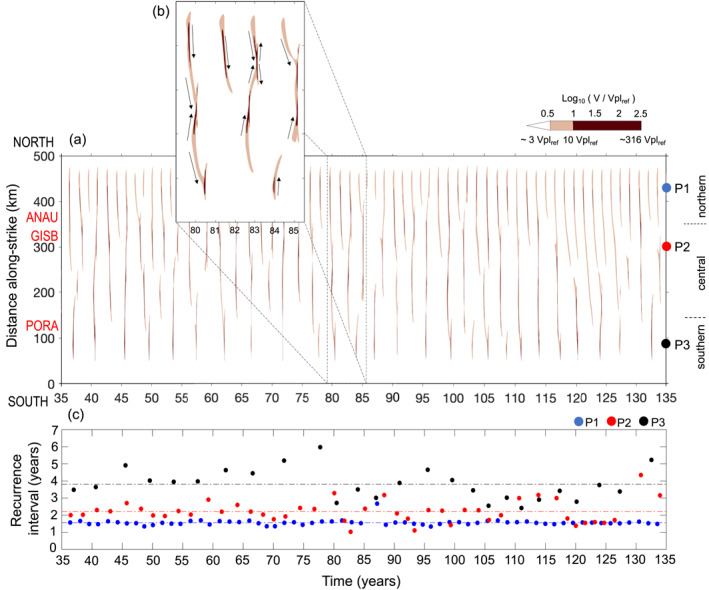
(a) Slip velocity evolution along the margin, in log_10_ V/Vpl_ref_ scale, at 10 km depth. Slip velocities larger than V $>\;10^{0.5}$ Vpl_ref_ (∼3 Vpl_ref_ or 0.39 mm/day) are plotted here. The entire range of slip velocities is shown in Figure S5 in Supporting Information S1. Red labels show along‐strike locations of some reference cGPS stations (see Figure 1 for location in map view). Colored circles indicate the along‐strike location of points P1, P2, and P3 shown in Figure 2a. Northern, central and southern indicate the three segments intro which the along‐strike distance is divided. (b) Zoom in of 6.5 yr. Dark arrows indicate the along‐strike migration of slip fronts. (c) Recurrence interval of SSEs [brown contours in (a)] at points P1, P2, and P3. Colored dashed horizontal lines indicate the average recurrence interval at each point.

To describe the long‐term slow slip behavior along the margin, we show the slip velocity at 10 km depth over 100 yr (Figure 5a and Figure S5 in Supporting Information S1). In this case, we assume a velocity threshold for SSEs of ∼3 Vpl_ref_ (10^−8.37^ m/s or 0.39 mm/day). Although this threshold is below the lower resolution limit of onshore GPS networks at Hikurangi (∼2 mm/day), it allows us to describe several features of the modeled SSEs. In Figure 5a, SSEs emerge as bands that occur periodically along‐strike (brown contours) and extend along most of the margin. Within each SSE, the slip rate can vary by a few orders of magnitude; high velocity patches (darker brown color in Figure 5b) are often linked up by regions of lower velocities (light brown color in Figure 5b). Some of these SSEs involve the interaction of multiple slip fronts that migrate along the fault (dark arrows in Figure 5b).

Modeled SSE recurrence intervals vary along the fault strike. We calculate the recurrence intervals of SSEs at three representative locations along the margin (P1, P2, and P3; Figure 5c). The average recurrence interval increases southward from 1.5 yr at P1 (dashed blue line in Figure 5c) to almost 4 yr at P3 (dashed black line in Figure 5c). This southward increase in the recurrence interval is broadly consistent with the observed pattern along the Hikurangi margin (Figure 1b). However, the recurrence intervals of modeled SSEs are slightly more variable in time during a given 20‐yr time interval than the observations (Figure 1b), especially in the southern part of the margin (P3 in Figure 5c).

Interestingly, peak slip rates at P1–P3 increase southward along‐strike (Figure S4b to S4d in Supporting Information S1), which correlates with the change in SSE recurrence intervals. The most frequent SSEs in the northern part of the margin have the lowest slip rates (Figure S4b in Supporting Information S1), whereas the least frequent SSEs in the south have the highest slip rates (Figure S4d in Supporting Information S1).

#### 3.2.2 SSE Source Properties

To analyze the misfit between our model and observed SSEs, we calculate the source properties of simulated SSEs (i.e., duration, magnitude, maximum slip, and maximum slip rate) and compare them with the observations. As source parameters depend on the resolution of onshore geodetic networks at Hikurangi, we assume a velocity threshold of 20 Vpl_ref_ (10^−7.5^ m/s or 2.46 mm/day), which is about the lower limit of resolved SSE velocities given in Ikari et al. (2020) catalog. SSE duration is defined as the time period over which the velocity threshold is exceeded. The corresponding SSE moment magnitude is calculated using the slip accumulated over the SSE duration and source area (defined as the region with slip greater than 2 cm). Note that we assume a shear modulus of 10 GPa to calculate the moment magnitude of simulated SSEs, consistent with the value used in Ikari et al. (2020) catalog, as well as estimates from Arnulf et al. (2021). Previous studies of Hikurangi SSEs assumed a 30‐GPa shear modulus when estimating the shallow SSE moments, and thus quoted larger *M*_w_ (*M*_w_ ∼ 7.0) for shallow Hikurangi SSEs (e.g., Wallace & Beavan, 2010; Wallace, Beavan, Bannister, & Williams, 2012, Wallace et al., 2016).

**Figure 6:**
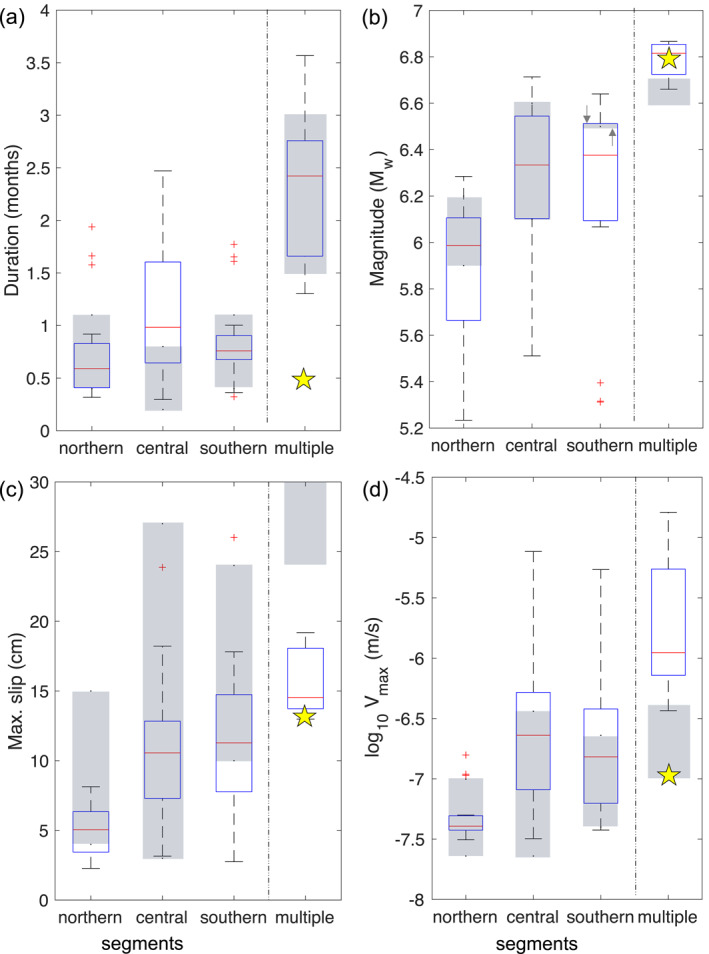
Modeled (box plot) and observed (gray‐shaded bars) source properties of SSEs that emerge at the northern, central, and southern segments. “Multiple” refers to multisegment SSEs. Gray‐shaded bars indicate the observed ranges of SSEs taken from the catalog in Ikari et al. (2020). According to the location of observed SSEs, we classified them into different segments. SSEs emerging offshore Tolaga Bay or North of Gisborne, were included in the northern segment; SSEs offshore Giborne, Mahia Peninsula or Hawke’s bay, in the central; and SSEs offshore Cape Turnagain in the southern. To constrain the range of multisegment SSEs, we consider the 2006 and 2011 SSE sequences as single SSEs, noting that each sequence is composed of several smaller SSEs that ruptured different segments along the margin. We then added up the moment and maximum slip of the smaller SSEs of each sequence, while the maximum slip rate corresponded to the largest velocity reached in each sequence. (a) Duration, (b) magnitude, (c) maximum slip, and (d) maximum slip rate are shown. Double arrows in (b) highlight the location of the observed range (gray‐shaded bar) in the southern segment. Blue box shows 50% of the simulated SSE source properties, from the 25th to the 75th percentile. Red line within the box corresponds to the median value. Dashed black line are the whiskers of the box, which cover ±2.7 times the standard deviation. Outliers are shown as red crosses. Yellow stars indicate the source properties of the 2016 East Coast SSE that was triggered by the Kaikoura earthquake’s seismic waves (Wallace et al., 2018).

We find that the simulated SSE source properties agree well with the observations in all three segments (Figure 6). In particular, the model results in relatively low slip rates and moment magnitudes of SSEs in the northern part of the margin, consistent with observations (Ikari et al., 2020; Todd & Schwartz, 2016). This overall agreement of the simulated and observed SSE source properties is remarkable, given the relatively simple model considered here. The model shows a slightly broader range of duration, magnitudes, and slip rates than those observed. This could be attributed to the longer time interval considered in the model (100 yr) compared to geodetic observations, which cover only the last ∼20 yr.

Seven synthetic SSEs ruptured more than one fault segment along‐strike at irregular periods over the 100 yr considered. Movie S2 shows an example of one multisegment SSE that ruptured both the southern and central segment. Compared to SSEs occurring in just one segment, multisegment SSEs have notably higher slip rate, magnitude and duration (multiple in Figure 6). We compare the source properties of multisegment SSEs with those of the 2016 East Coast SSE, triggered by the 2016 Kaikoura earthquake (Wallace et al., 2018), which ruptured ∼300 km along the Hikurangi margin (Wallace et al., 2017, 2018). The magnitude and maximum slip of the observed triggered SSE are within the modeled ranges (yellow star in Figure 6), yet the durations are overpredicted. This suggests that spontaneous SSEs may last longer than dynamically triggered SSEs do. Comparing multisegment SSEs with observed spontaneous (i.e., not triggered) SSEs that rupture more than one segment along‐strike (gray‐shaded bar for multiple in Figure 6), we see that the model reproduces well their magnitudes and durations, but not their maximum slip and slip rates. We note that there are only three multisegment SSEs observed at Hikurangi so far, and hence their source properties are not as well constrained as those of individual SSEs.

#### 3.2.3 Cumulative Slip of SSEs and Slip Budget

**Figure 7:**
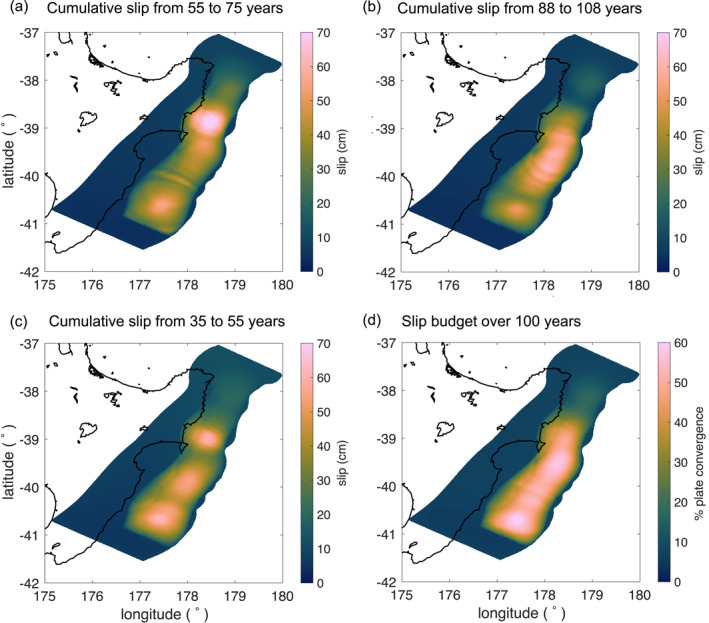
Cumulative slip of SSEs emerging in the preferred model from (a) 55–75 yr, (b) 88–108 yr, (c) 35–55 yr, and (d) Slip released by SSEs over 100 yr as a percentage of the plate convergence rate.

To determine the slip distribution of modeled SSEs over time, we sum the slip of SSEs that exceeded the velocity threshold (20 Vpl_ref_) within a given time period. Our results show that all the margin, between Cape Turnagain and East Cape, slips during SSEs; however, the specific cumulative slip distribution varies at a decadal scale. Figures 7a–7c show cumulative slip distribution over three 20‐yr time intervals. In Figure 7a, two large slip patches arise offshore Gisborne and Cape Turnagain with maximum cumulative slip of ∼70 and ∼50 cm, respectively. This pattern is qualitatively similar to the geodetic inversion of the cumulative slip distribution between 2002 and 2014 (Figure 1a), where two main slip patches develop at similar locations. At the same time, different cumulative SSE slip patterns emerge in other time intervals (Figures 7b and 7c). These results suggest that the slip distribution of shallow SSEs in Hikurangi may be variable over time.

To gain insight into the contribution of SSEs to the slip budget along the Hikurangi margin, we sum up the total cumulative slip released by SSEs over 100 yr and divide it by the total amount of slip accumulated due to plate convergence over the same period. Our results (Figure 7d) show that the fault releases up to 60% of the plate convergence via SSEs, with most of the slip released at the central and southern sections of the fault, offshore Mahia Peninsula and Cape Turnagain, respectively. Notably, this percentage decreases to ∼20% in the northern section of the fault (Figure 7d), north of Gisborne, despite SSEs being more frequent in that region. This difference is attributable to the relatively lower slip rates of SSEs in the northern part of the margin (Figure S4b in Supporting Information S1), as most of these events do not exceed the velocity threshold (20 Vpl_ref_), which causes the slip accumulated via SSEs to be comparatively low in that region. On the other hand, if we assume a velocity threshold of 3 Vpl_ref_, the percentage of slip released via SSEs is uniform within the SSE zone, from offshore East Cape to south of Cape Turnagain (Figure S6 in Supporting Information S1). In this case, SSEs release up to 90% of the slip accrued due to the plate convergence.

#### 3.2.4 Preferred Model With ${\bar{\sigma }}_{n}$ = 10 MPa in the SSE Zone

**Figure 8:**
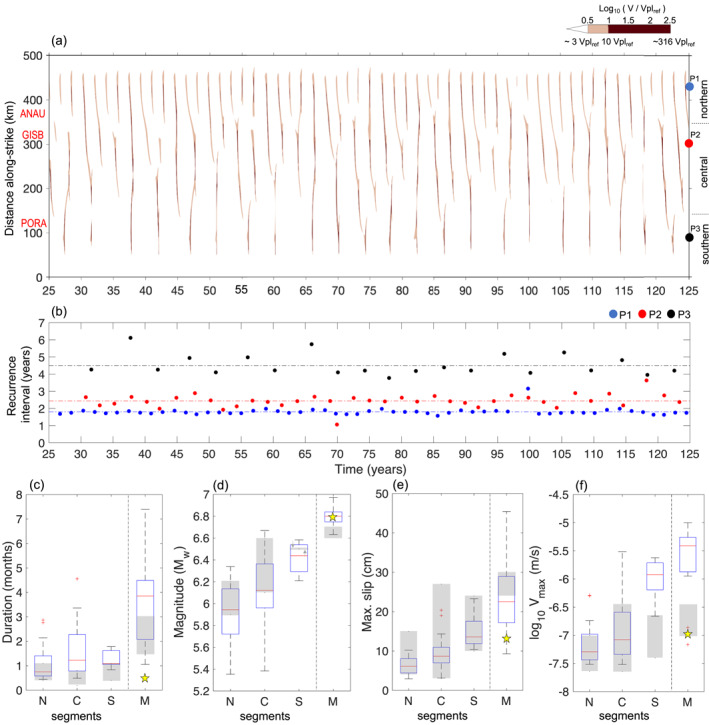
Preferred model for ${\bar{\sigma }}_{n}$ = 10 MPa and (*a *– *b*) = −0.000 3 in the SSE zone. (a) Slip velocity evolution along the margin, in log_10_ V/Vpl_ref_ scale, at 10 km depth. Slip velocities larger than 10^0^ ^.5^ Vpl_ref_ (∼3 Vpl_ref_ or 0.39 mm/day) are plotted here. (b) Recurrence interval of SSEs at points P1, P2, and P3 [colored circles in (a), see map view location in Figure 2a]. (c)–(f) Box plot shows the distribution of source properties of modeled SSEs at each segment. N, C, and S, stand for the northern, central, and southern segments, respectively. M denotes multisegment SSEs. Description of box plot is the same as in Figure 6. Gray‐shaded bars indicate observed ranges for SSE source properties, taken from Ikari et al. (2020) catalog.

We also select a preferred model among the simulations with ${\bar{\sigma }}_{n}$ = 10 MPa and (*a* − *b*)_vw_ = −0.000 3 in the SSE zone, following the same approach described in Section 3.1. The parameters of this model (orange arrow in Figures 3d–3f) are shown in Table 1. As in the preferred model with ${\bar{\sigma }}_{n}$ = 1 MPa and (*a* − *b*)_vw_ = −0.003, this model reproduces the main features of shallow SSEs reasonably well (Figure 8). In particular, the along‐strike segmentation of SSEs recurrence intervals are in good agreement with the observed pattern along Hikurangi (Figure 8b). On the other hand, modeled SSEs have slightly longer duration than observations (Figure 8c). The overall agreement between the models with a factor of 10 difference in ${\bar{\sigma }}_{n}$ suggests that the model results are not sensitive to ${\bar{\sigma }}_{n}$, but to the products $a{\bar{\sigma }}_{n}$ and $b{\bar{\sigma }}_{n}$, consistent with rate‐and‐state friction laws (Text S1 in Supporting Information S1).

#### 3.2.5 Alternative Model Setups

To investigate the effect of some of our modeling assumptions on the results, we consider three alternative model setups, referred to as Alternative Model A, B and C. A detailed description of each setup is given in the Text S2 in Supporting Information S1 and summarized in Table S1 in Supporting Information S1. For Alternative Model A, we consider an SSE zone that extends all the way to the trench (at 2.5 km depth), in contrast to the preferred model where the SSE zone starts at 4 km depth. This was motivated by the lack of constraints on the updip extent of shallow SSEs. For Alternative Model B, we assume a different parameter setting to better enforce the strongly locked condition in the southern part of the margin (0 km < *Y* < 50 km), which in our preferred model slides only slightly below the plate rate. For Alternative Model C, we assume that the SSE zone extends along the entire length of the fault along‐strike, thus we do not include the VW and VS bands on both sides of the model geometry, from 0 km < *Y* < 50 km and 475 km < *Y* < 500 km (Figure 2a), respectively. This model allows us to determine the effect of the parameter setting on the ends of the fault on SSE segmentation. The parameters chosen for each alternative model are the same as for the preferred model given in Table 1. We find that each alternative model reproduced the source properties of observed shallow SSEs (Figures S7–S10 in Supporting Information S1). Some differences exist between the model results, for instance in Alternative model C, SSEs occur along the entire fault along‐strike (i.e., 500 km). On the other hand, the along‐strike change in the recurrence interval is broadly consistent with observations along Hikurangi for all three alternative models (Figures S7b, S9b, and S10b in Supporting Information S1). These findings demonstrate that the overall fitness of our model is not significantly affected by these assumptions.

### 3.3 Controls on Along‐Strike Segmentation of SSE Recurrence Intervals

To investigate the main factors that control the segmentation of SSE recurrence intervals, we consider additional three different model setups M2–M4 (with M1 being the preferred model shown in Section 3.1). In M1, both the downdip width of the SSE zone (i.e., *W*) and the plate convergence rate vary along the strike of the fault (Section 2.2). To isolate the effect of a non‐planar fault and spatially variable plate convergence, we construct model M2 that has a uniform plate convergence rate along the margin, which is set to Vpl_ref_ (45 mm/yr), and Models M3 and M4 that have uniform *W* along‐strike with either variable (M3) or uniform (M4) plate convergence rate. To set *W* uniform along the margin, we use the planar fault geometry (Figures 2d–2f), described in Text S3 in Supporting Information S1. For M3 and M4, we assume the same model parameters given in Table 1, except that h* = 115 km and *d*_c_ = 10.2 mm. In this case, *W*/h* = 0.77, which is comparable to the value in M1 and M2, where *W*/h* ranges from 0.65 to 1.14 (for h* = 95 km). Ensuring similar values of *W*/h* for different simulation cases enables us to compare between model results without the influence of the differences in *W*/h*. Table S2 in Supporting Information S1 summarizes the characteristics of each model setup.

**Figure 9:**
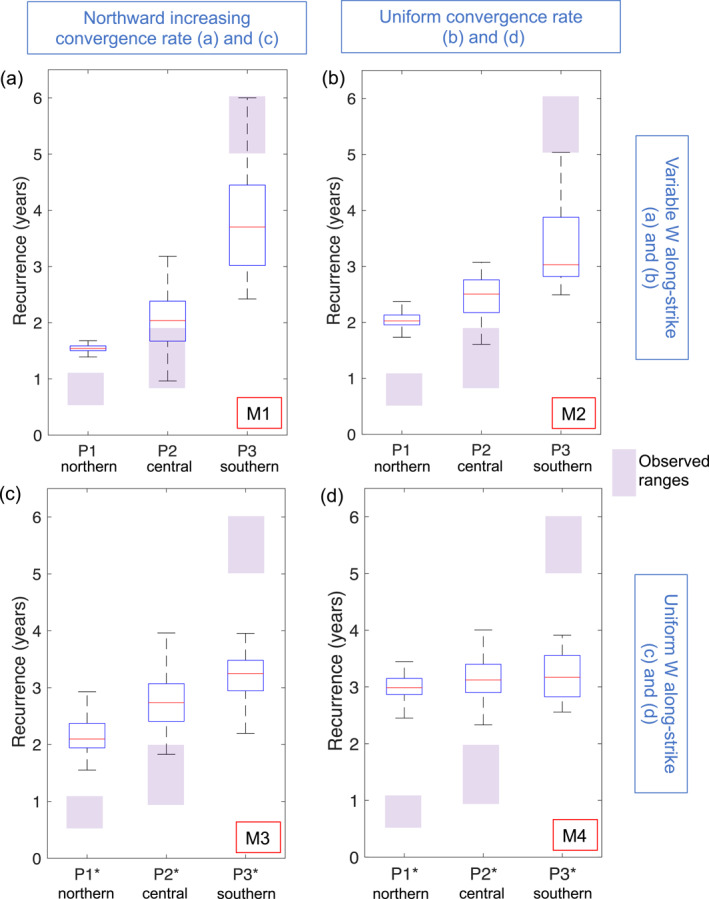
Recurrence interval of modeled SSEs at three points along the margin, P1^(^*^)^ to P3^(^*^)^ (see Figures 2a and 2d for location of points) over a 100‐yr period. Northern, central and southern correspond to the segments where each point is located. Purple‐shaded bars show the observed recurrence interval of SSEs estimated from Figure 1b. M1 corresponds to the preferred model described in Section 3.2. M2–M4 are additional models described in Section 3.3. Model setup with (a and b) non‐planar geometry and variable *W* along‐strike (Figure 2c), and with (c and d) planar geometry and uniform *W* along‐strike (Figure 2f). Simulations with (a and c) variable and (b and d) uniform plate convergence rate along‐strike. Box plots show the distribution of the recurrence intervals at each point. Blue box shows the distribution of 50% of the recurrence intervals, from the 25th to the 75th percentile. Red line within the box corresponds to the median value. Dashed black line are the whiskers of the box, which cover ±2.7 times the standard deviation. Outliers are not shown in this figure.

To determine the effect of the different model setups on SSE segmentation, we compare the along‐strike changes in the recurrence intervals of SSEs between these four models (M1–M4). To do so, we calculate the recurrence interval of peak slip rates that exceed 3 Vpl_ref_ at the same three locations along the margin (P1, P2, and P3 for the non‐planar fault and P1*, P2*, and P3* for the planar fault, red circles in Figures 2a and 2d, respectively). We find that for M1 and M3, the northward increase in the plate convergence rate correlates with the decrease in the recurrence interval along the margin (Figures 9a and 9c). The segmentation of the recurrence interval is still present in M2 (Figure 9b), but vanishes in M4 (Figure 9d). This suggests that along‐strike changes in *W* also contributes to the segmentation of the modeled SSEs. In particular, at P3, where *W* is the widest along the margin (Figure 2c), the recurrence intervals are the longest (Figure 9b), whereas the opposite is true for P1 (i.e., shortest recurrence interval and narrowest *W*), suggesting that *W* positively correlates with SSE recurrence intervals.

## 4 Discussion

### 4.1 Along‐Strike Segmentation of Shallow SSEs in Hikurangi

Our results suggest that the along‐strike change in the recurrence interval of shallow SSEs is controlled by spatial variations in both the downdip width of the SSE zone (i.e., model parameter *W*) and the plate convergence rate (*V*_pl_) along the margin. The inverse correlation between the plate convergence rate and SSE recurrence interval (Figures 9a and 9c) is consistent with both previous numerical results (Li et al., 2018; Shibazaki et al., 2012; Watkins et al., 2015) and the following simple analysis. For quasi‐periodic SSEs recurring every *T* years, the recurrence interval *T* can be expressed as $T=\Delta \tau /\dot{\tau }$, where Δ*τ* is the stress drop of quasi‐periodic SSEs of the same magnitude and $\dot{\tau }$ is an inter‐SSE stressing rate which would be proportional to *V*_pl_ (Kaneko et al., 2018). Since the stress drop of simulated SSEs are roughly constant, a faster convergence rate (larger *V*_pl_) would result in a shorter recurrence interval. Hence the northward increase of the convergence rate along the Hikurangi margin (Wallace et al., 2004) likely contributes to the shorter recurrence interval of SSEs offshore Tolaga Bay in the northeast, compared to Cape Turnagain, ∼300 km to the southwest.

In Section 3.3, we show that the recurrence interval of SSEs is also affected by spatially variable downdip width of the SSE zone (Figure 9). Assuming a uniform *W* along‐strike leads to SSEs with less segmented recurrence intervals (Figures 9c and 9d), while for variable *W* along‐strike, SSEs with longer recurrence intervals concentrate in the region with the widest *W* along the margin (Figures 9a and 9b). The positive correlation of *W* with SSE recurrence intervals is consistent with previous SSE models assuming both a 2D fault (Liu & Rice, 2009) and a 3D non‐planar fault (Shibazaki et al., 2012). The effect of *W* on shallow SSEs in Hikurangi could explain why their recurrence interval does not gradually increase along‐strike, as would be expected if only the plate rate influenced them (e.g., Figure 9c). Instead, an abrupt increase in the recurrence interval takes place from the central (∼1–2 yr) to the southern (∼5 yr) part of the margin (Figure 1b), coinciding with the change in *W* along‐strike (Figure 2c). Our results thus suggest that the effects of *W* and *V*_pl_ combine to enhance the segmentation of shallow SSEs. Although it is difficult to quantitatively assess which effect is dominant due to the nonlinearity of the model outcome, we note that the downdip width of the SSE zone appears to have a slightly stronger effect on the recurrence interval than the variable plate convergence rates, as variable *W* and uniform convergence rate leads to a stronger segmentation of the recurrence intervals than uniform *W* with variable plate convergence rates (compare Figures 9b and 9c).

Our results indicate that along‐strike change in the dip angle of the plate interface also contributes to the segmentation of SSEs along Hikurangi, as the downdip width of the SSE zone is inversely related to the plate dip angle. This explains why SSEs with longer recurrence interval concentrate in the southern part of the margin, where the plate dip angle is shallower (Barker et al., 2009). Our results support a previous numerical model of SSEs in Cascadia (Li & Liu, 2016) indicating that the dip angle influences the along‐strike segmentation of these events.

Our results do not rule out other potential factors that could affect shallow SSE segmentation along the Hikurangi margin. For instance, along‐strike changes in the effective normal stress have been linked to changes in the recurrence interval of simulated SSEs (Li et al., 2018; Liu, 2014). Along the Hikurangi margin, these changes are not well constrained, thus further research is required to determine whether this factor could contribute to SSE segmentation as well. It is also unclear whether changes in the thermal structure along the margin (Yabe et al., 2014) could also affect the periodicity of shallow SSEs at Hikurangi. In Section 4.5, we elaborate on other factors that were not considered in our modeling.

### 4.2 Effect of Spatial Variations in the Effective Fault Stiffness Ratio *W*/h* on SSE Properties and Fault Slip Behavior

The effective fault stiffness ratio *W*/h*, where *W* is the downdip width of the SSE zone and h* is the critical patch size to generate unstable slip (Rubin & Ampuero, 2005), has been shown to be a key parameter that controls both the fault slip behavior and the characteristics of SSEs (e.g., Barbot, 2019; Liu & Rice, 2005, 2007, 2009). Liu and Rice (2007) showed that the fault response transitions from decaying oscillations to seismic events with increasing *W*/h*, with slow slip emerging in between the two. Likewise, previous models of SSEs in 2D (Liu & Rice, 2009) and 3D non‐planar faults (Li & Liu, 2016; Perez‐Silva et al., 2021) pointed out that the source properties of SSEs (e.g., slip rate, recurrence interval and magnitude) tend to positively correlate with changes in *W*/h*.

Marked spatial variations of *W*/h* in our preferred model (Figure 2c, note that h* is constant along‐strike) have a notable effect on the source properties of SSEs, as longer recurrence intervals and larger slip rates correlate with larger *W*/h* (Figure 5c and Figure S4b–S4d in Supporting Information S1), and the converse is also true (i.e., shorter recurrence and lower slip rates correlate with lower *W*/h*). The fault slip response is also affected by changes in *W*/h*. Our parameter exploration shows that the slip behavior varies along the fault strike for most simulation cases (Figure 3). The difference in slip behavior at each fault segment is mainly controlled by the along‐strike change in *W*/h*; stable creep is the predominant slip pattern in the northern segment where *W*/h* is lowest, whereas SSEs mostly emerge in the southern segment, where *W*/h* is larger (Figure 3 and Figure S3 in Supporting Information S1). Similarly, most of the seismic events nucleate in the southern segment. Our results thus suggest that the effect of *W*/h* on SSE properties and fault slip behavior suggested by previous studies (e.g., Liu & Rice, 2005, 2009) may also hold for along‐strike variations of this parameter.

### 4.3 Implications for Megathrust Slip Behavior and SSE Environment in Hikurangi

We estimate that modeled SSEs offshore the east coast of the North Island release up to 60% of the plate convergence rate over 100 yr (Figure 7d), which suggests that SSEs are the main mechanism of strain release along the Hikurangi margin, consistent with geodetic inferences (Wallace & Beavan, 2010). We find that the estimation of the slip budget depends on the velocity threshold assumed to define SSEs; assuming a slip‐rate threshold about six times lower, modeled SSEs release up to 90% of the slip deficit (Figure S6 in Supporting Information S1). This result suggests that the resolution limit of GPS inversion models strongly influences the assessment of the contribution of SSEs to the total slip deficit. This is especially relevant in Hikurangi, where most of the slip during shallow SSEs concentrates offshore, away from the onshore geodetic network.

Our model suggests that shallow SSEs closely interact with each other along the Hikurangi margin. We see that both the initiation and arrest of an SSE usually involves the migration of slip fronts from or toward different regions along the fault (e.g., Figure 5b, Movie S1). Some SSEs occur simultaneously (SSE 2 and 3 in Figure 4) or spatially close to each other (SSE 1 and 2 in Figure 4). In other instances, two slip fronts merge into a single large SSE (Figures 4d and 4e), a behavior that is comparable to the coalescence of slow slip fronts observed in Cascadia (Bletery & Nocquet, 2020) and that has been linked to the initiation of earthquakes (Bletery & Nocquet, 2020; Kaneko & Ampuero, 2011). All this indicates that our simulated SSEs are typically not separated in time and space, thus they are likely to have strong stress interactions between each other (Liu, 2014). These stress interactions may influence the seismicity and tectonic tremor rates that accompany some shallow SSE sequences in Hikurangi (e.g., Bartlow et al., 2014; Jacobs et al., 2016; Kim et al., 2011; Romanet & Ide, 2019; Todd & Schwartz, 2016; Wallace, Beavan, Bannister, & Williams, 2012; Yarce et al., 2019).

Our model suggests that some shallow SSEs may rupture the whole margin along‐strike, as shown in Figure 5a. These events would comprise several subevents with faster slip (darker brown color in Figure 5b), which are linked up spatially by slower‐slipping regions. Although these whole‐margin SSEs have not been documented at Hikurangi, except for the SSE sequence triggered by the 2016 Kaikoura earthquake (Wallace et al., 2016), this lack of observations could be attributed to the limited resolution of the onshore geodetic network. These networks could only resolve the higher‐velocity patches, as seen in Figure 4 (SSEs 1–5), while the slower‐slipping regions in between these patches—where the slip rate is only slightly larger than ∼3 Vpl_ref_ (0.39 mm/day)—would be below the detection threshold (∼2 mm/day). For example, the observed shallow SSE sequence in 2011, where several SSEs of short duration (1–3 weeks) migrated northward along the margin over 6 months (Wallace, Beavan, Bannister, & Williams, 2012), could be attributed to a whole‐margin SSE of which only the high‐velocity patches were detected.

Previous modeling studies have assumed near‐lithostatic values of fluid pressure (${\bar{\sigma }}_{n}\;∼$1 MPa) in the source region of shallow SSEs in Hikurangi (Shibazaki et al., 2019; Wei et al., 2018). In contrast, our results suggest that the effective normal stress $\left({\bar{\sigma }}_{n}\right)$ could range from 1 to 10 MPa. The overall agreement between the models with a factor of 10 difference in ${\bar{\sigma }}_{n}$ (Figures 5, 6, and 8) suggests that pore fluid pressure in the SSE source region does not need to be a near‐lithostatic value. A sub‐lithostatic pore fluid pressure is also supported by full‐waveform inversion of active source seismic data offshore Cape Turnagain region, indicating 10–30 MPa in the shallow SSE source region (Arnulf et al., 2021).

### 4.4 SSE Source Scaling Relations

Scaling relations are often used to gain insight into the failure mechanism of SSEs, and how it differs from that of fast earthquakes. Based on a global compilation of slow earthquakes, the moment‐duration scaling of SSEs was originally proposed to follow a linear scaling (*M* ∼ *T*; Ide et al., 2007), which contrasts to the cubic scaling (*M* ∼ *T* ^3^) followed by earthquakes over a wide range of magnitudes (Kanamori & Anderson, 1975). Yet recent observations indicated a cubic moment‐duration scaling of SSEs in Cascadia (Michel et al., 2019), Nankai (Dal Zilio et al., 2020 using long‐term SSE catalog in Takagi et al., 2019), and Mexico (Frank & Brodsky, 2019) subduction zones, as well as in the San Andreas Fault (Tan & Marsan, 2020). Although it is still uncertain how well constrained these results are given the small sample size (e.g., 40 SSEs in Michel et al.’s, 2019 catalog) and the underlying assumptions when using LFEs as a proxy for SSEs (e.g., Frank & Brodsky, 2019).

**Figure 10:**
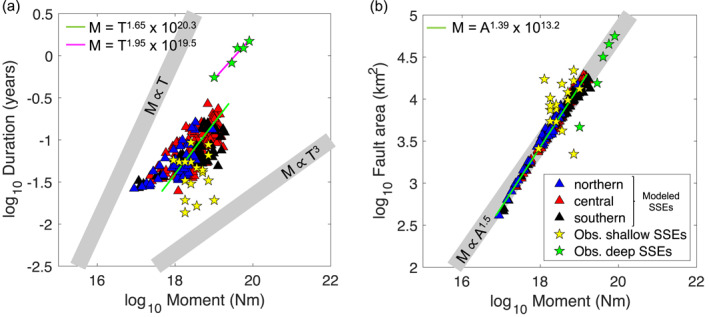
Comparison of scaling properties between observed (stars) and modeled (triangles) SSEs along the Hikurangi margin. Modeled SSEs are classified according to the segment: northern (blue), central (red), and southern (black). Source properties of observed SSEs (taken from Ikari et al., 2020 catalog) are classified into shallow (yellow stars) and deep (green stars) SSEs. (a) Moment‐duration scaling relation. Green line shows the best fit line for the modeled SSEs with *M* = *T* ^1.65^ × 10^20.3^. *M* ∝ *T* and *M* ∝ *T* ^3^ scalings are shown as reference. Magenta line shows the best fit line for observed deep SSEs with *M* = *T* ^1.95^ × 10^19.5^. (b) Moment‐area scaling relations. Green line shows the best fit line for the modeled SSEs with *M* = *A* ^1.39^ × 10^13.2^. *M* ∝ *A* ^1.5^ is shown as reference.

To determine the scaling relations of shallow SSEs, we take the Hikurangi SSE catalog of Ikari et al. (2020) and plot the moment (*M*) versus duration (*T*), and also versus area (*A*; Figure 10). We then compare these observed source properties with those of the simulated SSEs in our preferred model described in Section 3. We find that the source properties of observed shallow SSEs (yellow stars in Figure 10) broadly overlap with those of simulated SSEs (triangles in Figure 10), further validating our model. As expected, the observed source properties of deep SSEs (green stars in Figure 10) show larger moments and duration than the shallow SSEs. The observed source properties of shallow SSEs in Hikurangi do not show a clear trend, which could be due to a limited range of durations and moments sampled by the shallow SSEs, as well as a short catalog (<20 yr). Unlike their shallow counterparts, deep SSEs follow a distinguishable quadratic trend of the moment with respect to duration, with a best‐fit scaling of *M* = *T* ^1.95^ × 10^19.5^ (magenta line in Figure 10a). A larger catalog is needed to determine whether this scaling trend still holds.

The scaling trends of simulated shallow SSEs are clearer than for the observed shallow events. The best‐fit moment‐area relation of simulated SSEs follows *M* ∼ *A* ^1.39^ (Figure 10b), which is close to the best fit exponent of 1.5 found in previous models of SSEs (Dal Zilio et al., 2020; Liu, 2014). On the other hand, the moment of simulated SSEs scales with the duration with an exponent of 1.65 (Figure 10a), although the scattering of the triangles makes it hard to define a clear trend. This scaling relation is comparable to *M* ∼ *T* ^1.3^ found by previous SSE models (Liu, 2014; Shibazaki et al., 2012); our relatively larger exponent could be attributed to the interaction between slip fronts along the margin (Figures 4 and 5b), which as shown by Liu (2014) leads to a scaling exponent closer to 2. To test the sensitivity of our results to the velocity threshold, we calculated the scaling relations assuming two additional thresholds (i.e., 15 Vpl_ref_ and 25 Vpl_ref_) and find only slight differences in the scaling exponents (<± 0.15; Figure S11 in Supporting Information S1). We hypothesize that the fact that the moment‐duration scaling of our simulated SSEs (*M* ∼ *T* ^1.65^) falls in between the previously suggested cubic and linear scalings could indicate that the scaling properties of SSEs are probably less clear‐cut than previous studies have suggested. If true, it would suggest that factors such as the source depth of SSEs or their tectonic environment could also affect their scaling relations.

### 4.5 Model Limitations

Our modeling approach involves several assumptions and simplifications. As a first approximation, we assume that the frictional properties at the source depths of shallow SSEs are spatially homogeneous. However, rock‐friction experiments using material entering the SSE source region at the Hikurangi margin indicate that the spatial distribution of frictional properties is likely more complex, as input sediments exhibit contrasting lithological (Barnes et al., 2020) and frictional properties (Boulton et al., 2019; Rabinowitz et al., 2018). Future modeling studies may account for frictional heterogeneity by modeling patches of VW and VS materials (Luo & Liu, 2021; Skarbek et al., 2012; Yabe & Ide, 2018), or by implementing a relative strength ratio that accounts for the proportions of these materials (Barnes et al., 2020; Boulton et al., 2019; Luo & Ampuero, 2018).

Our model geometry represents a smooth plate interface and ignores the geometric complexity in the region where shallow SSEs occur along the Hikurangi margin. Such complexity has been imaged by active source seismic studies offshore Gisborne, where significant relief (>2 km) and roughness of the basement surface (Barnes et al., 2020), and the presence of seamounts (Bell et al., 2010) have been inferred. These findings together with the fact that several shallow SSEs in other subduction zones are also associated with rough plate interfaces (Saffer & Wallace, 2015; Wang & Bilek, 2014) suggests that accounting for smaller‐scale roughness may play an important role in the generation mechanism of shallow SSEs (Romanet et al., 2018; Sun et al., 2020). To account for such geometrical complexity, future models may include normal stress variations (e.g., Romanet et al., 2020), which are not considered in our present model as we assume a smooth plate interface.

Following the classic rate‐and‐state friction formulation (e.g., Dieterich, 1979), our model assumes that friction parameter (*a* − *b*) and the characteristic slip distance (*d*_c_) are independent of the sliding velocity. In contrast, laboratory measurements on drill samples from different subduction zones, including Hikurangi, show a systematic variation of (*a* − *b*) and *d*_c_ with slip velocity (Boulton et al., 2019; Ikari et al., 2009; Ikari & Saffer, 2011; Rabinowitz et al., 2018). A recent numerical model accounting for this slip‐rate dependence of (*a* − *b*) and *d*_*c*_ successfully reproduced SSEs characteristics over a broader range of parameters than with the classic rate‐and‐state formulation (Im et al., 2020). This could explain why the parameter range that can reproduce SSEs comparable to observations is relatively narrow in our model (Section 3.1).

Our modeling approach assumes that the effective normal stress is independent of time at the source depths of shallow SSEs, an assumption commonly invoked by numerical models of SSEs (e.g., Liu & Rice, 2009; Matsuzawa et al., 2013; Shibazaki et al., 2012, 2019). Yet recent observations in Nankai (Nakajima & Uchida, 2018), Cascadia (Gosselin et al., 2020), Mexico (Frank et al., 2015), and Hikurangi (Warren‐Smith et al., 2019; Zal et al., 2020) subduction zones inferred temporal changes of pore fluid pressure and hence the effective normal stress during and between SSEs. These changes are attributed to a fault valving behavior (Sibson, 1990, 1992) that possibly results from cyclical permeability changes induced by slip during SSEs (Gosselin et al., 2020; Nakajima & Uchida, 2018; Warren‐Smith et al., 2019; Zal et al., 2020). Future modeling work accounting for fluid valving behavior in simulations of Hikurangi SSEs is needed.

## 5 Conclusions

We have investigated the cause of along‐strike changes in the source properties of shallow SSEs along the Hikurangi margin using numerical simulations of fault slip that incorporate rate‐and‐state friction laws and a non‐planar fault geometry. Our model reproduces the magnitude and duration of shallow SSEs, as well as the segmentation of their recurrence intervals, which increase southward along the strike of the margin.

Our model results indicate that along‐strike variations in both the plate convergence rate, and the downdip width of the region of low effective normal stress and VW frictional properties (or SSE zone), play an important role in the segmentation of SSE recurrence intervals along the Hikurangi margin. We find that a wider SSE zone and a lower plate convergence rate favor SSE cycles with long recurrence interval. This could explain why shallow SSEs offshore Cape Turnagain, where the plate convergence rate and the SSE zone are respectively lower and wider than further north along strike, have longer recurrence interval (∼5 yr) than elsewhere along the margin (1–2 yr). Moreover, the shallow dipping angle of the plate interface in this portion of the margin contributes to a wider downdip width of the SSE zone, which indicates that along‐strike variations in the plate geometry also promote the segmentation of these events.

Our results show that the cumulative slip distribution of modeled SSEs is variable over a decadal scale, as SSE slip patches concentrate at different locations along the margin at different time intervals. This result suggests that slip distribution of shallow SSEs along Hikurangi may also vary in the future.

We have found that models assuming either ${\bar{\sigma }}_{n}$ = 1 or 10 MPa in the SSE zone reproduce the main features of shallow SSEs in Hikurangi. These results imply that ${\bar{\sigma }}_{n}$ need not be as low as 1 MPa at the source depths of shallow SSEs, contrary to the assumptions of several previous modeling studies.

## Supporting information

Supporting Information S1

Movie S1

Movie S2

## Data Availability

The data used to produce the figures is available at https://doi.org/10.5281/zenodo.5574236. The simulation code used in this study was developed by Li and Liu (2016).
