# Supplementary material for: Segmentation of Shallow Slow Slip Events at the Hikurangi Subduction Zone Explained by Along‐Strike Changes in Fault Geometry and Plate Convergence Rates
Source: J Geophys Res Solid Earth. 2022 Jan 21;127(1):e2021JB022913. doi: 10.1029/2021JB022913 (PMC9285732; doi:10.1029/2021JB022913)
Supplement: Supplementary file 1 — Supporting Information S1 [file JGRB-127-0-s003.pdf]

# Supporting Information for "Segmentation of shallow slow slip events at the Hikurangi subduction zone explained by along-strike changes in the fault geometry and plate convergence rates"

Andrea Perez-Silva[1], Yoshihiro Kaneko[2], Martha Savage[1], Laura Wallace[3,4],

Duo Li[5], Charles Williams[3]

[1]School of Geography, Environment and Earth Sciences, Victoria University of Wellington, PO Box 600, Wellington 6140, New Zealand.

[2]Department of Geophysics, Kyoto University, Sakyo-ku, Kyoto 606-8501, Japan.

[3]GNS Science, PO Box 30368, Lower Hutt 5011, New Zealand.

[4]Institute for Geophysics, University of Texas at Austin, Austin, TX, USA.

[5]Department of Earth and Environmental Sciences, Ludwig-Maximilians-University Munich, Theresienstrasse 41, 80333 Munich, Germany.

## Contents of this file

1. Text S1 to S3
2. Captions for Movie S1 and S2
3. Figures S1 to S11
4. Tables S1 to S2

## Additional Supporting Information (Files uploaded separately)

1. Movie S1 and S2

**Text S1: Governing equations**

The quasi-dynamic formulation describes the relation between the stress and the slip history on the fault (Rice, 1993). This formulation is an approximation of the fully dynamic equations, in that it does not account for the full inertial (wave) effects, i.e. for the stress changes due to wave propagation (Rice, 1993). Instead, these changes are approximated by a radiation damping term, that represents the final static stress changes as predicted by the exact solution of the full elastodynamic equations (Rice, 1993). The spatial and temporal discretization of this formulation is given by:

$$\tau_i(t) = -\sum_{j=1}^{N} K_{i,j}(\delta_j(t) - V_{pl}t) - \eta \frac{d\delta_i(t)}{dt} \ , \tag{1}$$

where $t$ is the time step and the subscripts $i, j$ are associated with an individual cell. $\tau_i$ and $\delta_i$ are shear stress and slip at element $i$, respectively. $V_{pl}$ is the plate convergence rate, which in our model setup increases northwards along the fault strike from 36 to 60 mm/yr (Figure S1). The term $\eta$ represents the radiation damping factor, defined as $\eta = \frac{\mu}{c_s}$, where $\mu$ is the elastic shear modulus and $c_s$ is the shear wave speed. The stiffness matrix or Green's function, $K_{i,j}$, describes the change in shear stress on element $i$ due to a unit dislocation in the dip direction on element $j$. $K_{i,j}$ is calculated in an elastic half-space medium and adapted to triangular dislocation elements by Stuart, Hildenbrand, and Simpson (1997). We note that the model neglects the effect of ocean bottom topography, i.e. it is assumed flat. This assumption is reasonable given the relatively small length scale of ocean bottom topography ($<$ 3 km) compared to the lengths of target SSEs ($\sim$50 km to $\sim$150 km).

The code incorporates rate- and state-dependent frictional (RSF) laws (Dieterich, 1979), in which the shear strength, $\tau$, is described as a logarithmic function of the slip rate $V$ and a state

variable $\theta$, which represents the temporal state of the asperity contacts and time dependent processes (Blanpied et al., 1998). The constitutive law follows the equation:

$$\tau = \bar{\sigma}_n f = \bar{\sigma}_n [f_0 + a \ln\left(\frac{V}{V_0}\right) + b \ln\left(\frac{V_0 \theta}{d_c}\right)], \tag{2}$$

where $f$ refers to the instantaneous friction coefficient, $f_0$ is the steady state friction coefficient at reference rate $V_0$ and $d_c$ is the characteristic slip for state evolution. $\bar{\sigma}_n$ is the effective normal stress, defined as the difference between the lithostatic stress and pore fluid fluid pressure ($\bar{\sigma}_n = \sigma_n$ - $p$). $a > 0$ and $b > 0$ are constitutive parameters that represent the instantaneous change of friction due to a sudden change in velocity and the evolution of friction with slip distance, respectively (Dieterich, 1979). Parameter $(a - b)$ determines the frictional stability regime of the fault, when $(a - b) > 0$, steady-state friction $f_{\mathrm{ss}}$ increases with velocity, known as steady-state velocity-strengthening (VS). In a VS regime, slip is always stable. A steady-state velocity-weakening (VW) regime occurs when $(a - b) < 0$. In this regime, slip could be unstable (seismic) or conditionally stable (Scholz, 1998). Friction parameter $(a - b)$ depends on the temperature, the rock type and the effective normal stress (Marone et al., 1990; Blanpied et al., 1998).

In our model, the evolution of the state variable is described by the Dietrich or 'aging' law, which assumes that the state variable and friction evolve during stationary contacts (Dieterich, 1979):

$$\frac{d\theta}{dt} = 1 - \frac{V\theta}{d_c}. \tag{3}$$

At steady state, the state variable can be interpreted as the lifetime of contact areas ($d_c/V_0$), assuming that $d_c$ is a typical contact size (Ampuero & Rubin, 2008).

Other formulations of the evolution of the state variable have been proposed. In the Ruina or 'slip law', the evolution of the state variable always involves slip, even during stationary contacts (Marone et al., 1990). Composite laws, that combine several versions of RSF law, have also been proposed (Kato & Tullis, 2001). The formulation that best describes a range of laboratory experiments remains a subject of ongoing research (Bhattacharya et al., 2017; Kaneko et al., 2016).

A theoretical estimate of the upper bound of a critical nucleation size is given by Rubin and Ampuero (2005):

$$h^* = \frac{2\mu b d_c}{\pi(1-\nu)(b-a)^2\bar{\sigma}_n}, \tag{4}$$

where $\mu$ and $\nu$ are the shear modulus and the Poisson ratio, respectively. $d_c$ is the characteristic slip distance, $\bar{\sigma}_n$ the effective normal stress and $(b-a)$ is the average value of the friction parameter in the region under VW conditions. In this study, we assume $\mu = 15$ GPa and $\nu = 0.25$.

**Text S2: Alternative model setups**

Apart from the model setup presented in the main text, we consider three alternative setups (A, B and C) to examine the consequence of some of our modeling assumptions. To assess the fitness of each alternative setup, we compare the source properties of simulated SSEs with observations, following the same approach describe in the main text (Section 3.2.2). Note that the model parameters for the three alternative models are the same as in the preferred model (Table 1).

*Alternative Model A*: In this model, we consider the case of an SSE zone extending all the way to the trench, at 2.5 km depth below sea level. This setup differs from the preferred model, where the SSE zone starts at 4 km depth, and was motivated by the lack of constraints on the updip limit of slip of SSEs. To keep the value of $h^*$ as in the preferred model, we slightly move the

downdip limit of the SSE zone upwards. The slip rate evolution along depth (at Y = 103 km) for Alternative Model A and the preferred model are shown in Figure S8. In Model A, during SSE episodes, larger slip rates ($V > 10\ Vpl_{ref}$) extend all the way to the trench (brown contours in Figure S8d), whereas in the preferred model, slip rates in the trench region increase only slightly above the plate convergence rate ($Vpl_{ref} < V < \sim 1.8\ Vpl_{ref}$) during SSEs (light brown contours in Figure S8b). In contrast, the slip rate evolution along-strike (Figure S7a) is similar to that in the preferred model (Figure S5). Model A reproduces the along-strike segmentation of SSEs (Figure S7b), as well as their source properties (Figure S7c-f). Based on these results, we cannot rule out that observed SSEs could also extend all the way to the trench.

*Alternative Model B*: To better enforce the strong coupling inferred in the southern part of the margin (Wallace & Beavan, 2010), in this model we assume a different parameter setting in this region. Following a similar approach to Liu and Rice (2007), we reduce the value of $d_c$ in the region from 0 to 50 km along-strike, such that $h^*$ is the same in the coupled region as in the SSE zone (i.e. 95 km). We find that this new setup leads to slip velocities of at least one order of magnitude lower than $Vpl_{ref}$ for $0\ km < Y < 50\ km$, as shown in Figure S9a. Over time the region gradually unlocks; for instance at 110 years the plate slides close to the plate convergence rate between 0 km to 25 km along-strike (Figure S9a). This model setup captures the along-strike segmentation in the recurrence interval of shallow SSEs (Figure S9b), as well as their source properties (Figure S9c-f), which indicates that the locking condition does not significantly affect the model results.

*Alternative Model C*: In this case we do not consider the VW and VS bands on both ends of the model geometry, from $0\ km < Y < 50\ km$ and $475\ km < Y < 500\ km$, respectively. Instead, we assume that the SSE zone also extends across these regions. This setup was motivated to

determine whether the segmentation of SSEs depended on the specific parametrizations of these regions. The slip rate evolution along-strike (Figure S10a) indicates that assuming this model setup, SSEs extend across the entire model geometry along-strike (from 0 < Y < 500 km) within the SSE zone. This contrasts with the preferred model, where SSEs extend from 50 < Y < 475 km. At the same time, we find that, despite the longer spatial extent of SSEs due to the larger SSE zone, the recurrence interval of these SSEs is still segmented along-strike (Figure S10b), which indicates that the slip behavior on the boundaries of the preferred model does not affect the segmentation of these events.

**Text S3: Planar fault geometry**

In section 3.3, we consider a planar fault to investigate the importance of non-planar fault geometry on the segmentation of modeled SSEs. The planar fault geometry has the same along-strike length and depth range as the non-planar geometry does, with the difference that the fault dip angle is constant ($\alpha = 7\ ^\circ$). We discretize the planar fault by 21607 triangular elements using Trellis software, each triangle has an area of ~3.9 km$^2$ and a side length ($dx$) of ~3 km. In this case, we assume a larger cell size than in the non-planar geometry to reduce computational costs, however, this difference does not affect the numerical resolution of the model, as we ensure that $h^*$ is well resolved. In this setup $h^* = 115$ km, thus $h^*/dx > 30$, which is larger than the ratio assumed by Liu and Rice (2005) in their planar fault model, where $4 < h^*/dx < 8$.

**Movie S1: Slip rate evolution on the fault over several SSE cycles:** The movie shows the slip velocity on the fault over the time interval shown in the snapshots of Figure 4. See Section 3.2.1 for a description of the slip rate evolution.

**Movie S2: Slip rate evolution during a multisegment SSE.** The movie shows an example of a multisegment SSE that ruptures the southern and central part of the fault. The event

nucleates in the southern part of the margin (offshore Cape Turnagain) and splits into two divergent slip fronts. The northward-propagating slip front migrates at a speed of ∼2.4 km/day. When approaching Mahia Peninsula the SSE reaches the maximum slip velocity, $V_{max}$ $\sim 10^{-6}$ m/s. Afterwards, it splits again into two divergent slip fronts. We note that this event was considered a single event, instead of two consecutive ones, because the slip velocity exceeds the velocity threshold of 20 $\mathrm{Vpl_{ref}}$ over the total duration of the event.

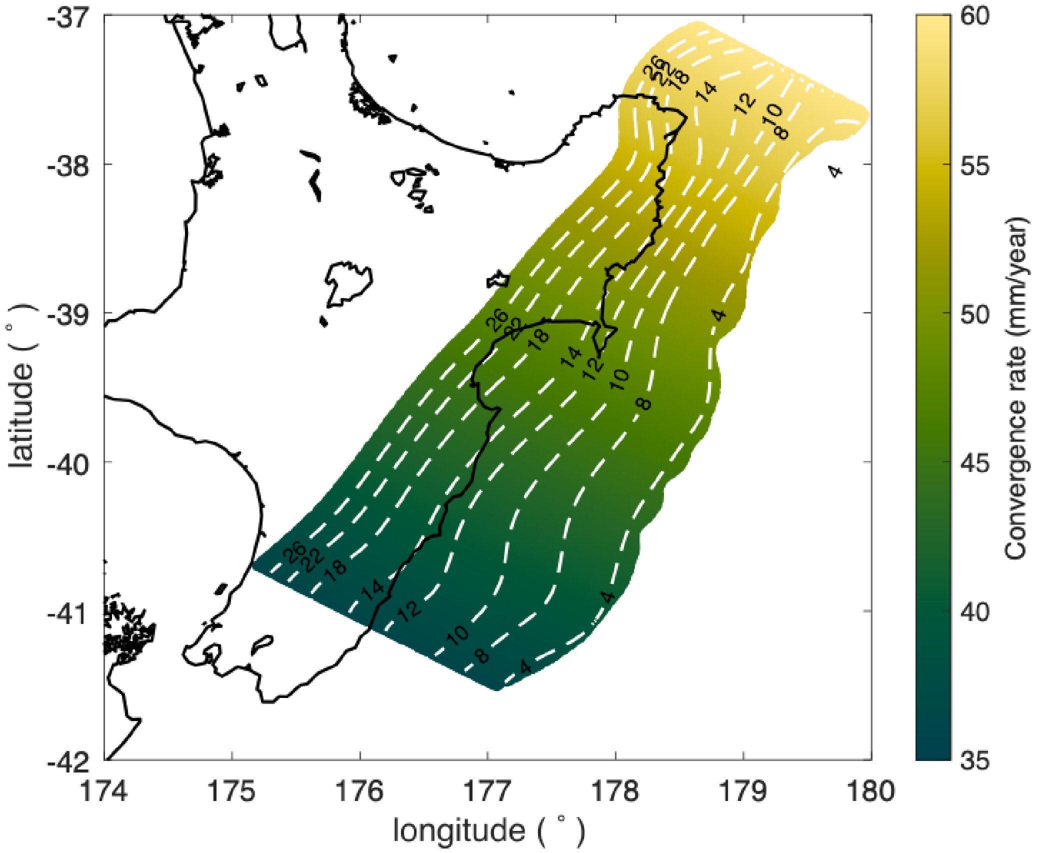


**Figure S1.** 3D non-planar geometry of the Hikurangi plate interface based on Williams et al. (2013). Dashed white lines represent isodepth contours in km. The plate convergence rate increases from 36 to 60 mm/yr along the strike of the model geometry, following the estimates in Wallace et al. (2004).

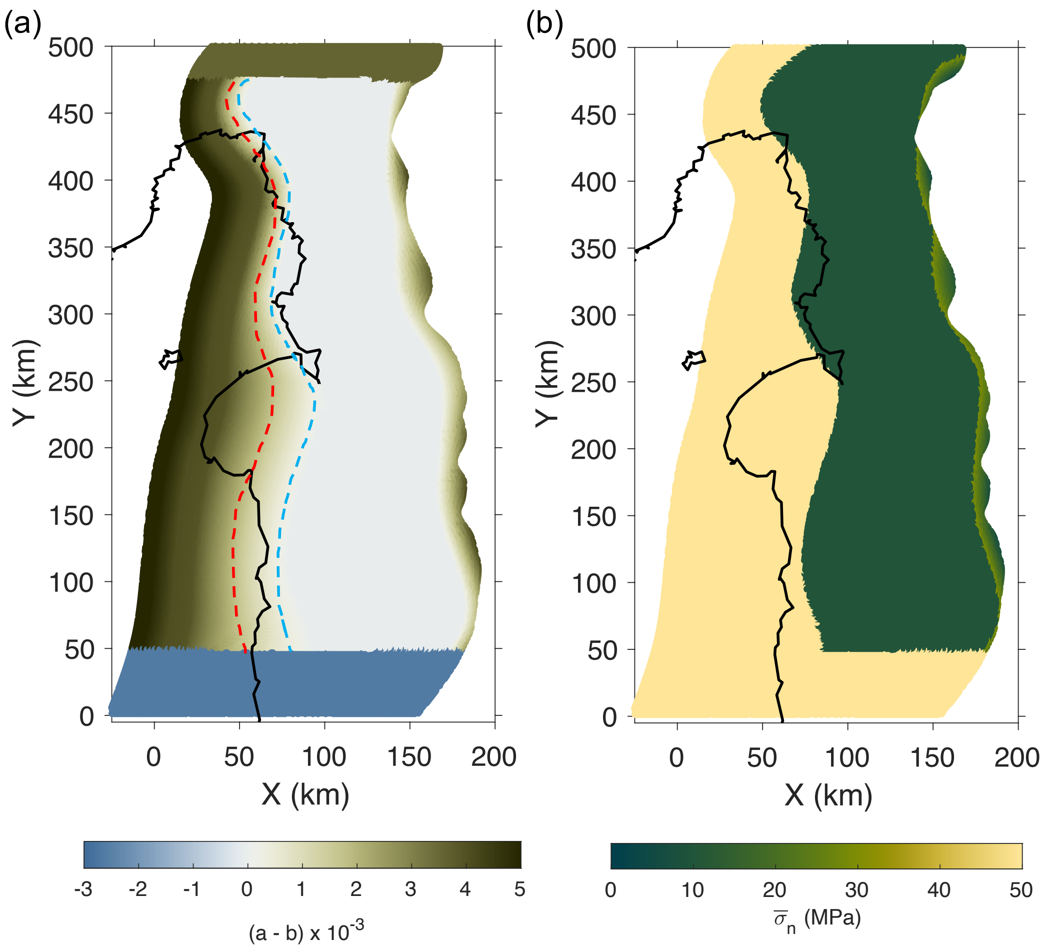


**Figure S2.** Model setup for simulations with $\bar{\sigma}_n$ = 10 MPa and $(a-b)_{\rm vw}$= -0.0003 in the SSE zone. Map view distribution of (a) friction parameter $(a-b)$ and (b) $\bar{\sigma}_n$ on the fault. Note that because we assume a linear increase of $(a-b)$ from VW to VS region, the location of the downdip limit of the VW-VS transition between the models with $(a-b)_{\rm vw}$ = -0.003 and $(a-b)_{\rm vw}$ = -0.0003 is slightly different; the former (dashed red line in (a)) is slightly deeper than the latter (dashed blue line in (a)). This difference does not affect the along-strike distribution of $W$, as the VW-region under low $\bar{\sigma}_n$ is the same in both model setups.

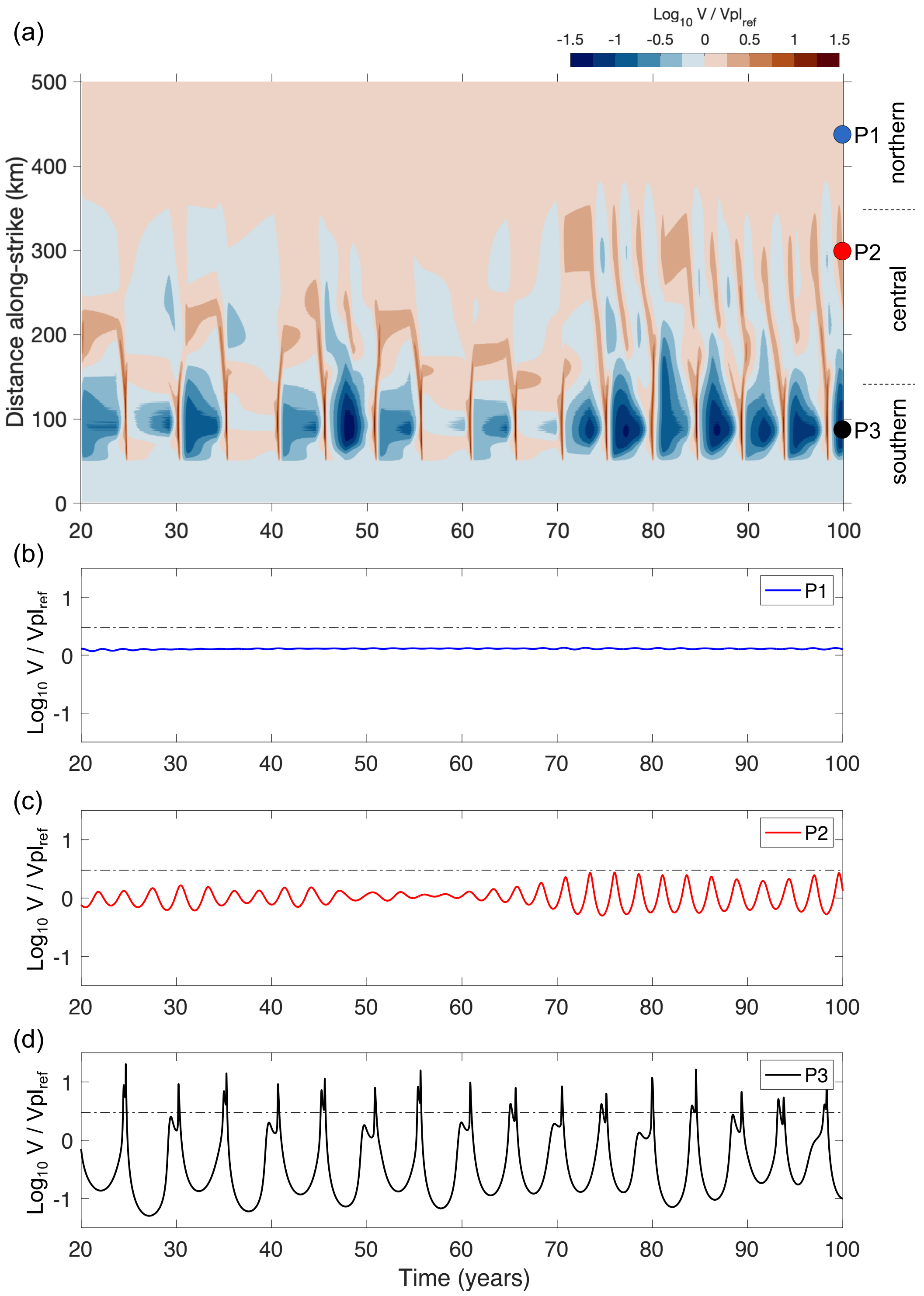


**Figure S3.** Example of simulation with different slip patterns along-strike. Simulation case with $a/b$=0.75, $W_{ave}/h^* = 0.5$ and $(a-b)_{\mathrm{vw}}$ = -0.0003. (a) Slip velocity evolution along the margin, in $\log_{10}$ V/Vpl$_{\mathrm{ref}}$, at 10 km depth. Colored circles highlight three reference points (P1, P2 and P3). (b)-(c) Slip rate at three points along the margin (P1, P2 and P3). Dashed-dotted line indicates 3 Vpl$_{\mathrm{ref}}$. The northern segment exhibits stable creep behavior, whereas in the southern segment SSEs emerge periodically. In the central segment, slip behavior is transitional from stable creep to SSEs.

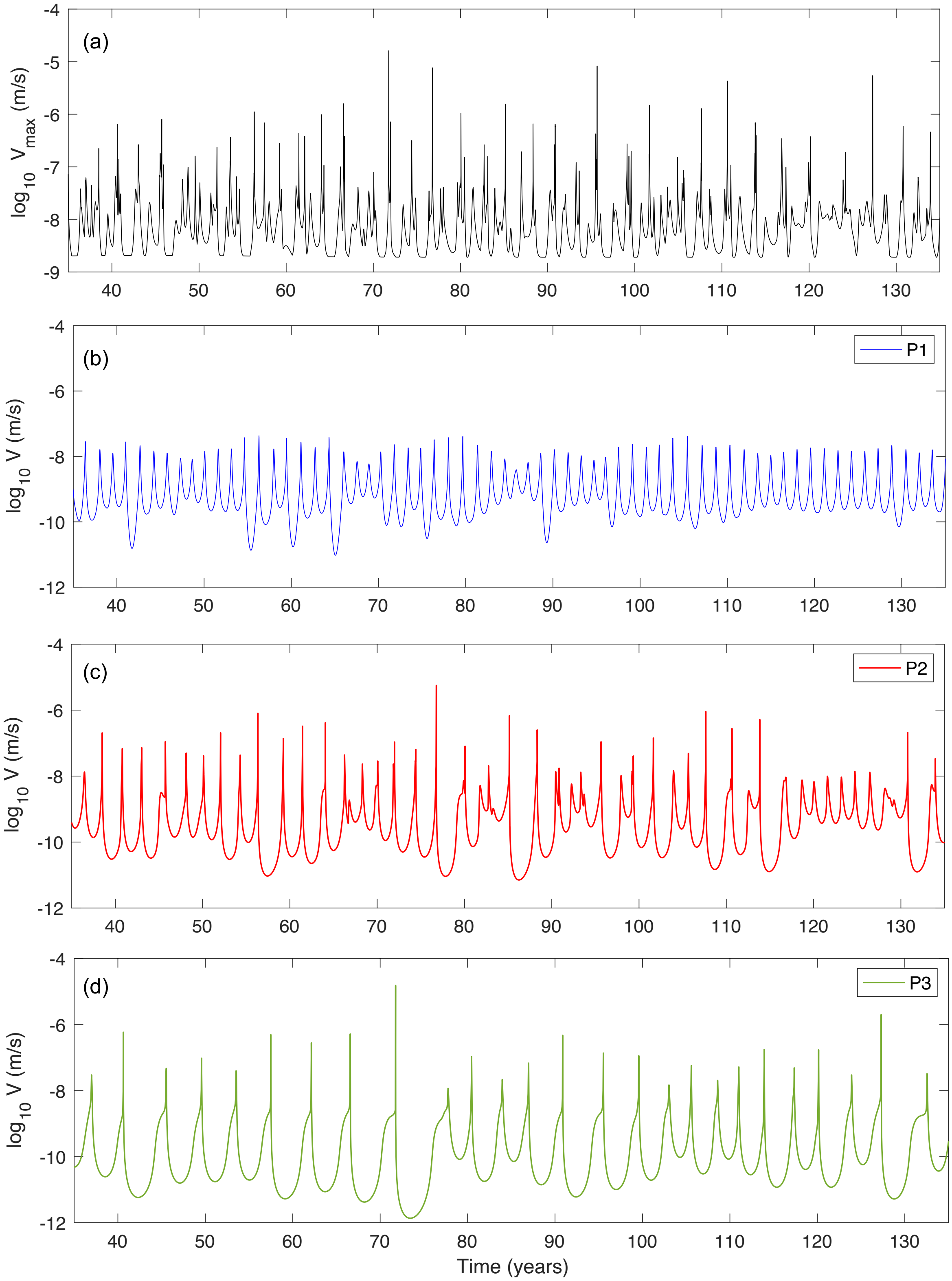


**Figure S4.** Slip rate during a 100-year simulation period from preferred model. Maximum slip rate along the fault (a). Slip rate at points P1 (b), P2 (c) and P3 (d) (located in Figure 2a). Peak slip velocities, as well as the time interval between peak velocities, increase from P1 to P3.

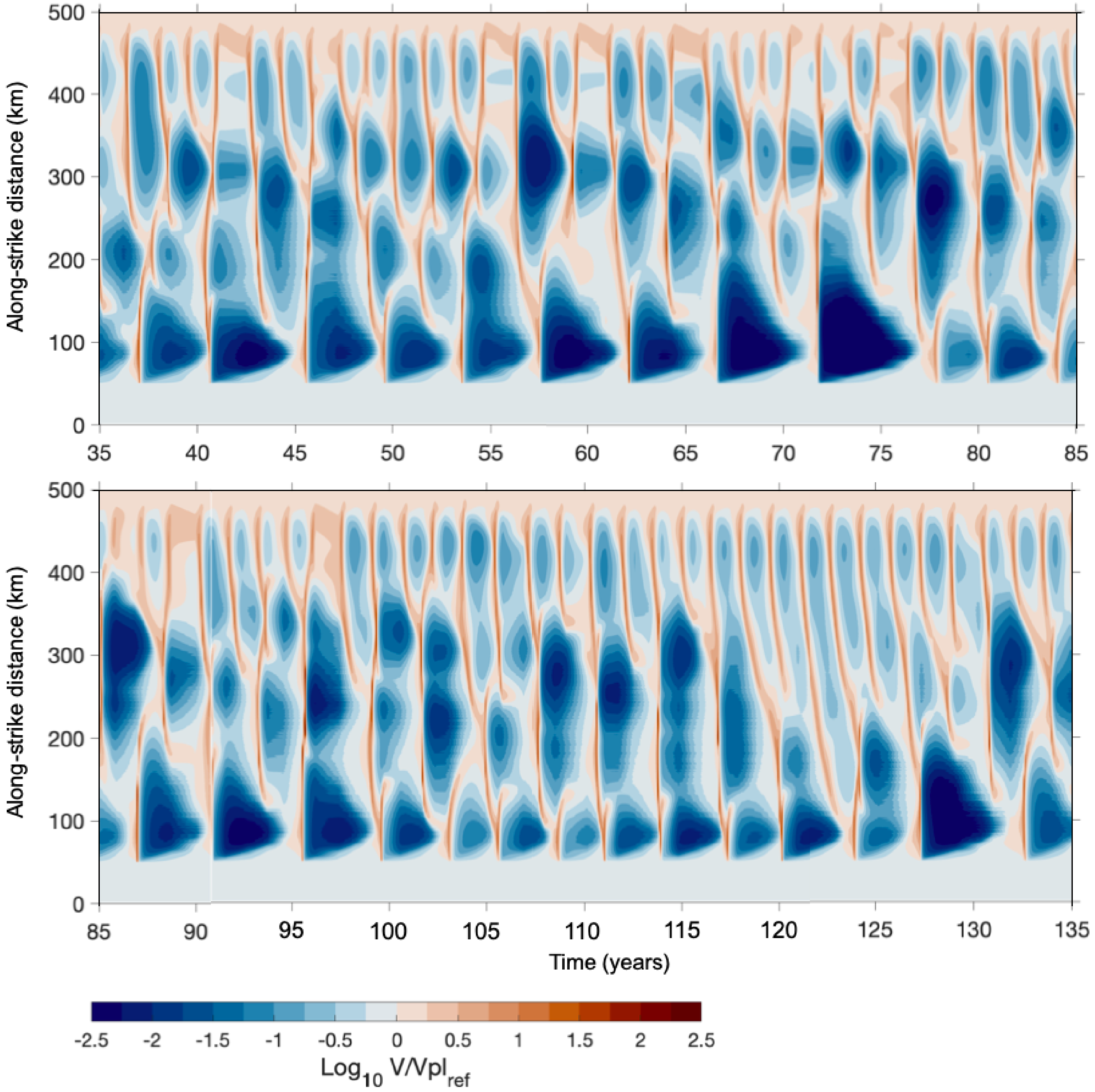


**Figure S5.** Evolution of slip velocity along strike, in $\log_{10}$ ($V/Vpl_{ref}$ ) scale, at 10 km depth. Results correspond to the preferred model.

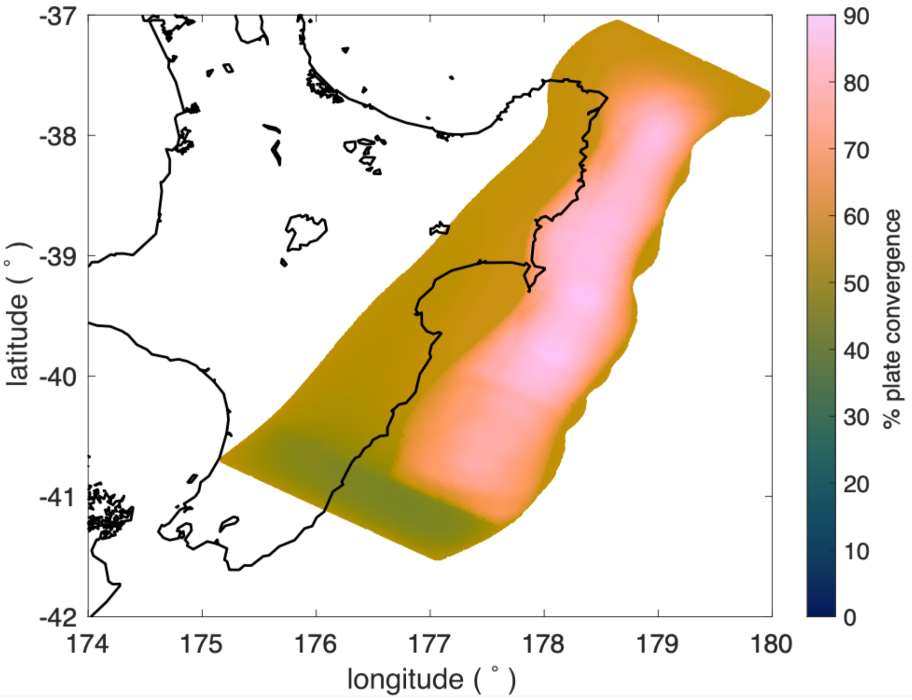


**Figure S6.** Slip released by SSEs over 100 years as a percentage of the plate convergence rate assuming a slip threshold of 3 $Vpl_{ref}$ to define an SSE. Results correspond to the preferred model.

**Table S1.** Summary of alternative model setups (A, B, C) described in Text S2.

| Alternative model setups | Model setup* | Model results* |
|---|---|---|
| Model A (Figs. S7, S8) | The SSE zone extends all the way to the trench at 2.5 km depth below sea level | Larger slip velocities (V > 10 $Vpl_{ref}$) reach the trench |
| Model B (Fig. S9) | Different parameter setting in the southern part of the margin (for 0 km<Y <50 km) to better enforce the strongly locked coupling | The plate slides up to one order of magnitude below the plate rate in the southern part of the margin (0 km <Y <50 km) |
| Model C (Fig. S10) | No VW nor VS bands at the ends of the model geometry along-strike | SSEs extend along the entire fault along-strike (500 km) |

* We only describe the differences with respect to the preferred model in the main text (M1).

**Table S2.** Description of models M1-M4 presented in Section 3.3.

| Models | Fault geometry | Model parameters | W along the fault strike | Plate rate ($V_{pl}$) along the fault strike |
|---|---|---|---|---|
| M1 (preferred, Figs. 4-7, 9a, 10) | Non-planar | Given in Table 1. $\bar{\sigma}$ and $(a-b)$ distribution given in Figs 2a to 2b | W varies along-strike (Figure 2c) | $V_{pl}$ increases northward along-strike (Figure S1) |
| M2 (Fig. 9b) | Non-planar | Same as M1 | Same as M1 | $V_{pl} = 45$ mm/yr everywhere |
| M3 (Fig. 9c) | Planar | Given in Table 1, except that $d_c =$ 10.2 mm and $h^* =$ 115 km. $\bar{\sigma}$ and $(a-b)$ distribution given in Figures 2d to 2e | W is uniform along-strike (Figure 2f) | $V_{pl}$ increases northward along-strike |
| M4 (Fig. 9d) | Planar | Same as M3 | Same as M3 | $V_{pl} = 45$ mm/yr everywhere |

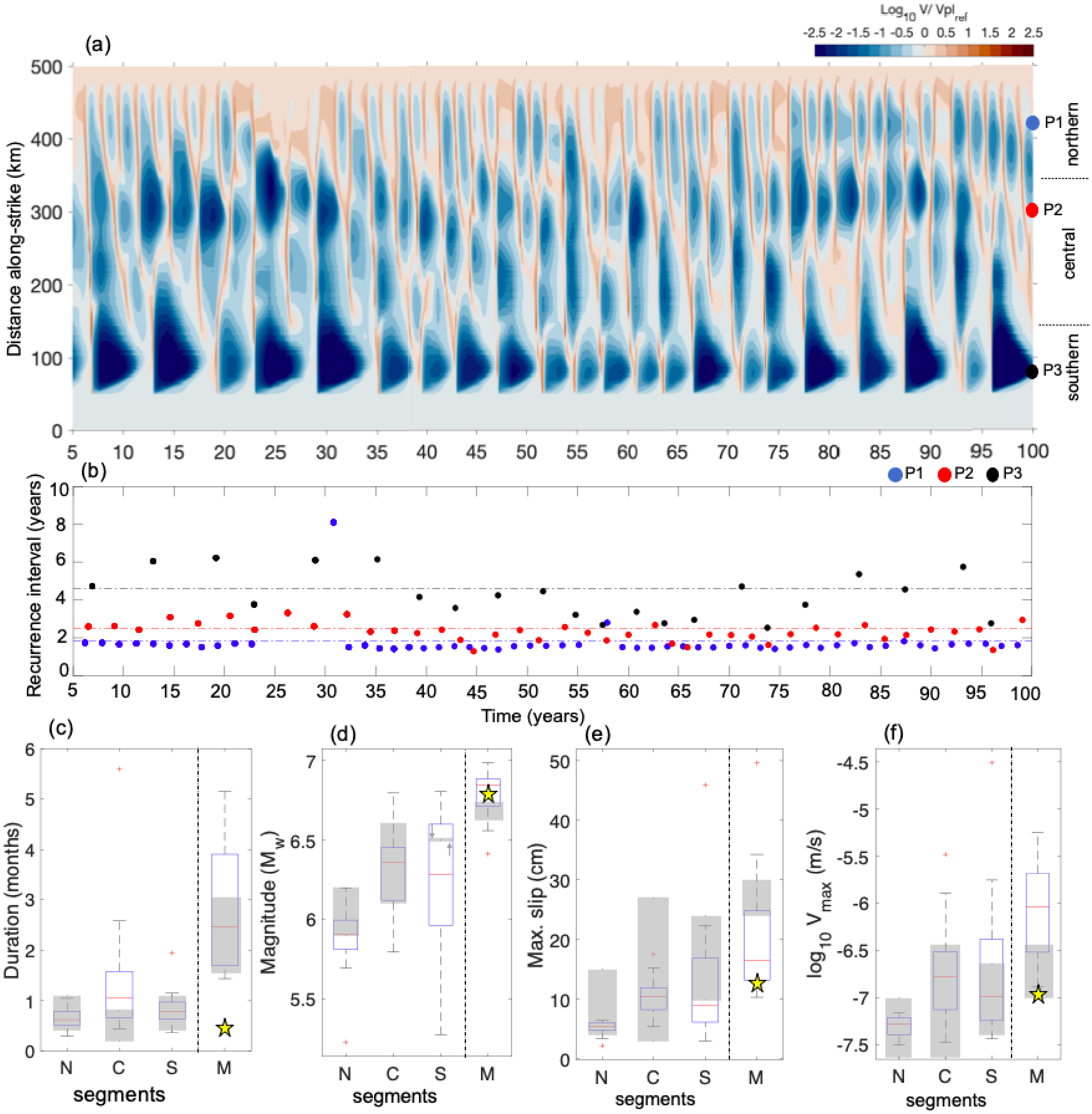


**Figure S7.** Results for *Model A*. Simulation case with SSE zone starting from the trench at 2.5 km depth. (a) Slip rate along-strike, in $\log_{10}$ V/V$pl_{ref}$, at 10 km depth. (b) Recurrence interval of slow slip episodes at points P1, P2 and P3 (colored circles in item (a), see map view location in Figure 2a). (c-f) Box plot show the distribution of source properties of modeled SSEs at each segment. N, C, S correspond to northern, central and southern segments. M denotes multisegment SSEs. Gray bars indicate observed ranges for SSEs' source properties taken from Ikari et al. (2020) catalog. Box plot description is the same as for Figure 6 in the main text.

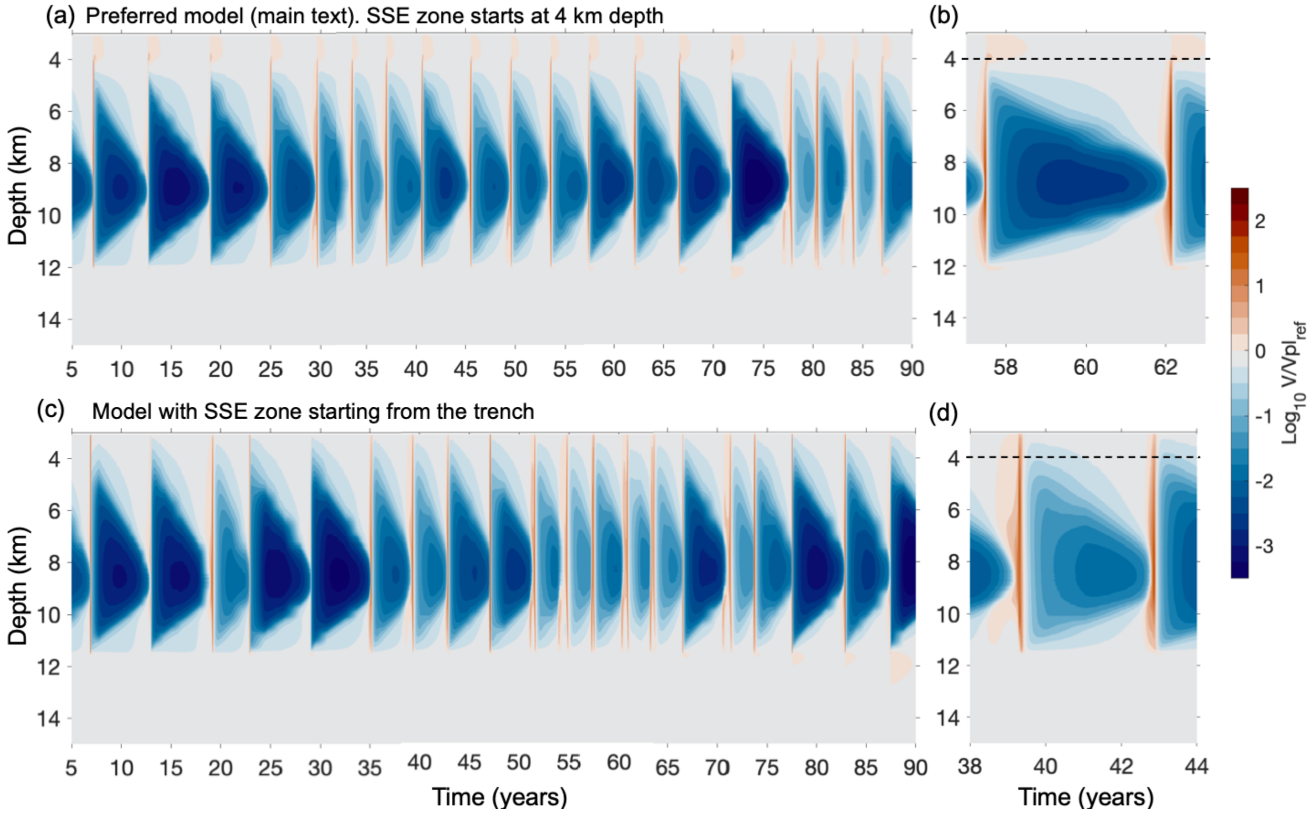


**Figure S8.** Slip rate evolution along depth at Y = 103 km for (a) Preferred model with SSE zone starting from 4 km depth. (b) Zoom in over six years from item (a). Dashed line highlights 4 km depth. (c) Model B with SSE zone starting from the trench, at 2.5 km depth. (d) Zoom in over six years from item (c). For model in (c), larger slip rates, (brown contours where V > 10 $Vpl_{ref}$) extend all the way to the trench, whereas in the preferred model only slip rates close to the plate rate ($Vpl_{ref} < V < 10^{0.25}$ or $\sim$1.8 $Vpl_{ref}$, light brown contours) reach the trench.

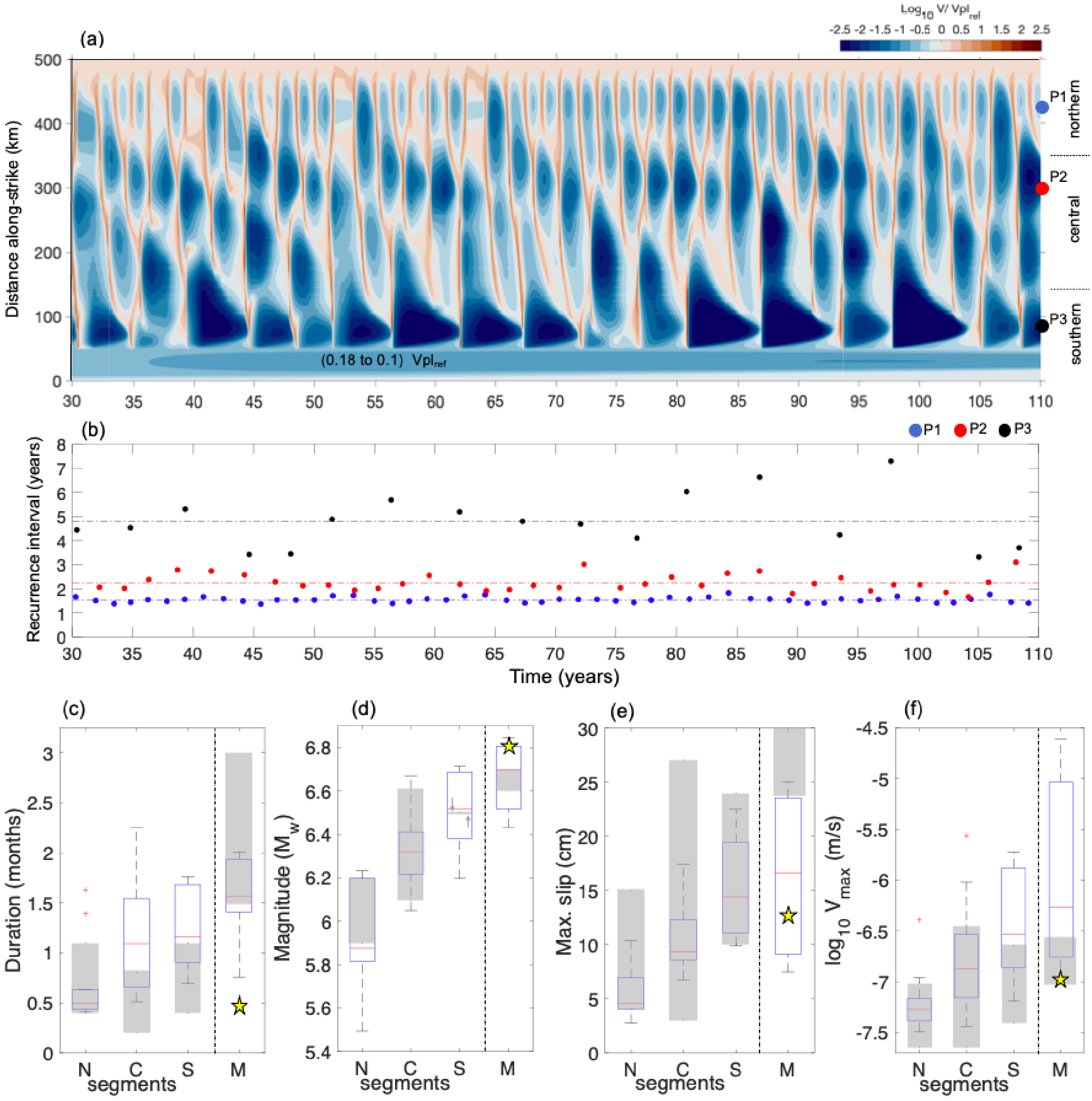


**Figure S9.** Results for *Model B*. Simulation case with different parametrization in the southern part of the fault (0 km < Y < 50 km) to better model the strongly locked region. (a) Evolution of slip velocity along strike, in $\log_{10}$ ($V/Vpl_{ref}$) scale, at 10 km depth. The slip rate in the southern part of the margin are in the range of 0.18 to 0.1 $Vpl_{ref}$ after ∼35 years, although the velocity gradually increases over time. (b) Recurrence interval of slow slip episodes at points P1, P2 and P3 (colored circles in item (a), see map view location in Figure 2a). (c-f) Box plot shows the distribution of source properties of modeled SSEs at each segment. N, C, S correspond to northern, central and southern segments. M denotes multisegment SSEs.

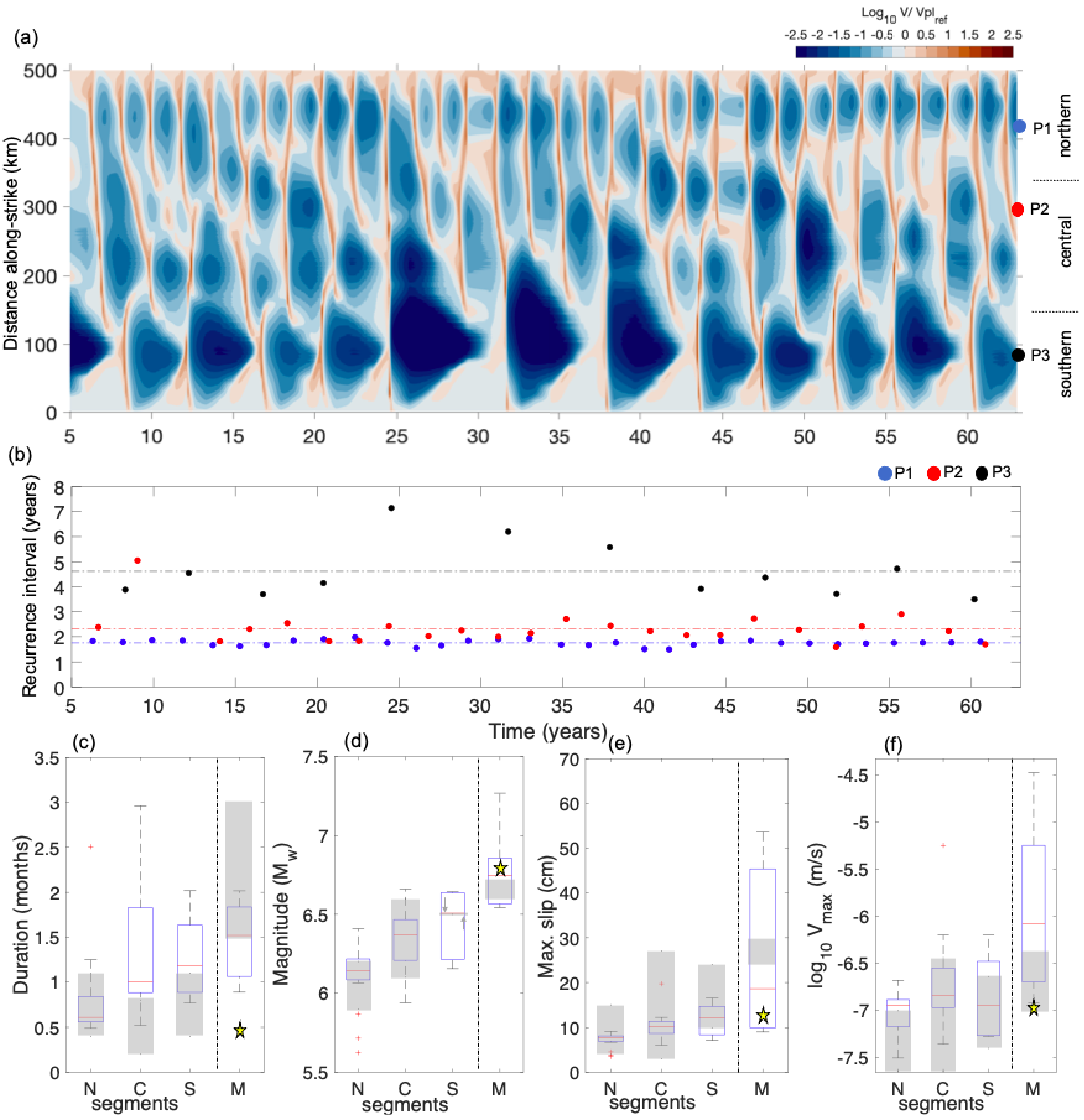


**Figure S10.** Results for *Model C*. Simulation case without VS and VW bands on the northern and southern ends of the model geometry, respectively. (a) Evolution of slip velocity along strike, in $\log_{10}$ ($V/Vpl_{ref}$) scale, at 10 km depth. (b) Recurrence interval of slow slip episodes at points P1, P2 and P3 (colored circles in item (a), see map view location in Figure 2a). (c-f) Box plot shows the distribution of source properties of modeled SSEs at each segment. N, C, S correspond to northern, central and southern segments. M denotes multisegment SSEs. Gray bars indicate observed ranges for SSEs' source properties taken from Ikari et al. (2020) catalog. Box plot description is the same as for Figure 6 in the main text.

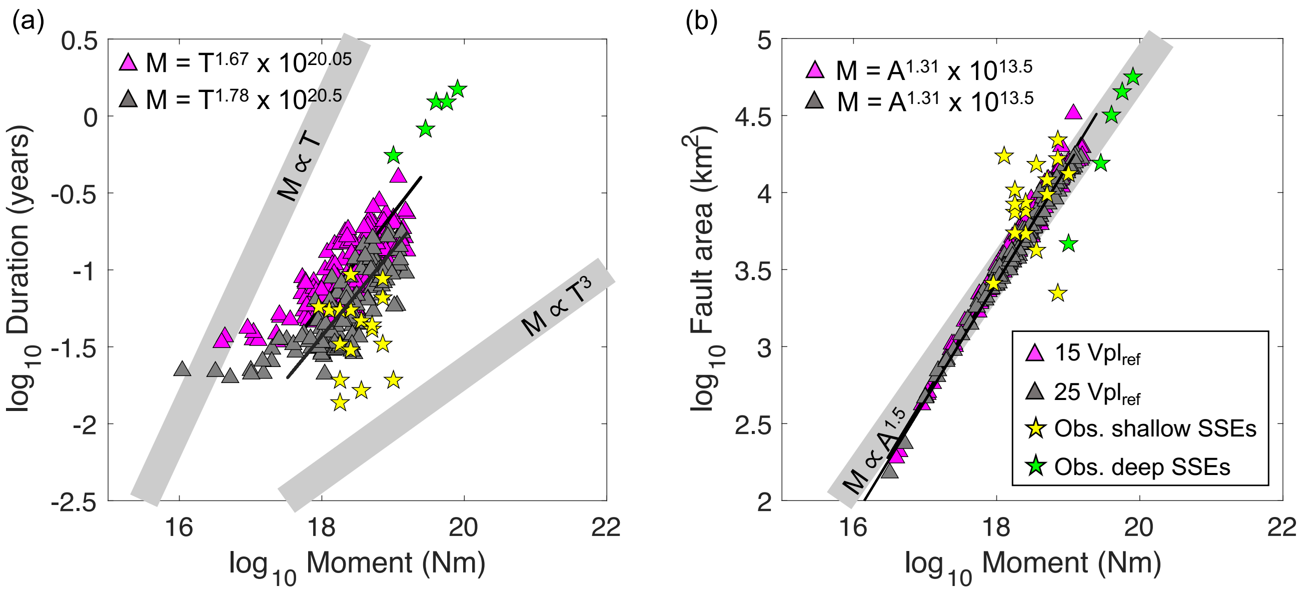


**Figure S11.** Scaling relations for modeled SSEs (triangles), assuming two different velocity thresholds: 15 $Vpl_{ref}$ or 1.85 mm/day (magenta triangles) and 25 $Vpl_{ref}$ or 3.08 mm/day (gray triangles). Source properties from observed shallow (yellow stars) and deep (green stars) SSEs (taken from Ikari et al. (2020) catalog) are included for comparison. (a) Moment-duration scaling. $M \propto T$ and $M \propto T^3$ scaling are shown as reference. (b) Moment-area scaling. $M \propto A^{1.5}$ is shown as reference. Best fit scaling for simulated SSEs shown as black line and given on top of the figure for each velocity threshold. Results correspond to the preferred model described in the main text.

## References

Ampuero, J.-P., & Rubin, A. M. (2008). Earthquake nucleation on rate and state faults–Aging and slip laws. *Journal of Geophysical Research: Solid Earth*, *113*, B01302. doi: 10.1029/2007JB005082.

Bhattacharya, P., Rubin, A. M., & Beeler, N. M. (2017). Does fault strengthening in laboratory rock friction experiments really depend primarily upon time and not slip? *Journal of Geophysical Research: Solid Earth*, *122*(8), 6389–6430. doi: 10.1002/2017JB013936.

Blanpied, M., Marone, C., Lockner, D., Byerlee, J., & King, D. (1998). Quantitative measure of the variation in fault rheology due to fluid-rock interactions. *Journal of Geophysical Research: Solid Earth*, *103*(B5), 9691–9712. doi: 10.1029/98JB00162.

Dieterich, J. H. (1979). Modeling of rock friction: 1. Experimental results and constitutive equations. *Journal of Geophysical Research: Solid Earth*, *84*(B5), 2161–2168. doi: 10.1029/JB084iB05p02161.

Ikari, M. J., Wallace, L. M., Rabinowitz, H. S., Savage, H. M., Hamling, I. J., & Kopf, A. J. (2020). Observations of laboratory and natural slow slip events: Hikurangi subduction zone, New Zealand. *Geochemistry, Geophysics, Geosystems*, *21*(2), e2019GC008717. doi: 10.1029/2019GC008717.

Kaneko, Y., Nielsen, S. B., & Carpenter, B. M. (2016). The onset of laboratory earthquakes explained by nucleating rupture on a rate-and-state fault. *Journal of Geophysical Research: Solid Earth*, *121*(8), 6071–6091. doi: 10.1002/2016JB013143.

Kato, N., & Tullis, T. E. (2001). A composite rate-and state-dependent law for rock friction. *Geophysical Research Letters*, *28*(6), 1103–1106. doi: 10.1029/2000GL012060.

Liu, Y., & Rice, J. R. (2005). Aseismic slip transients emerge spontaneously in three-dimensional

rate and state modeling of subduction earthquake sequences. *Journal of Geophysical Research: Solid Earth*, *110*, B08307. doi: 10.1029/2004JB003424

Liu, Y., & Rice, J. R. (2007). Spontaneous and triggered aseismic deformation transients in a subduction fault model. *Journal of Geophysical Research: Solid Earth*, *112*, B09404. doi: 10.1029/2007JB004930.f.

Marone, C., Raleigh, C. B., & Scholz, C. (1990). Frictional behavior and constitutive modeling of simulated fault gouge. *Journal of Geophysical Research: Solid Earth*, *95*(B5), 7007–7025. doi: 10.1029/JB095iB05p07007.

Rice, J. R. (1993). Spatio-temporal complexity of slip on a fault. *Journal of Geophysical Research: Solid Earth*, *98*(B6), 9885–9907. doi: 10.1029/93JB00191.

Rubin, A. M., & Ampuero, J.-P. (2005). Earthquake nucleation on (aging) rate and state faults. *Journal of Geophysical Research: Solid Earth*, *110*, B11312. doi: 10.1029/2005JB003686.

Scholz, C. H. (1998). Earthquakes and friction laws. *Nature*, *391*(6662), 37–42. doi: 10.1038/34097.

Stuart, W. D., Hildenbrand, T. G., & Simpson, R. W. (1997). Stressing of the New Madrid seismic zone by a lower crust detachment fault. *Journal of Geophysical Research: Solid Earth*, *102*(B12), 27623–27633. doi: 10.1029/97JB02716.

Wallace, L. M., & Beavan, J. (2010). Diverse slow slip behavior at the Hikurangi subduction margin, New Zealand. *Journal of Geophysical Research: Solid Earth*, *115*, B12402. doi: 10.1029/2010JB007717.

Wallace, L. M., Beavan, J., McCaffrey, R., & Darby, D. (2004). Subduction zone coupling and tectonic block rotations in the North Island, New Zealand. *Journal of Geophysical Research: Solid Earth*, *109*, B12406. doi: 10.1029/2004JB003241.

Williams, C. A., Eberhart-Phillips, D., Bannister, S., Barker, D. H., Henrys, S., Reyners, M., & Sutherland, R. (2013). Revised interface geometry for the Hikurangi subduction zone, New Zealand. *Seismological Research Letters*, *84*(6), 1066–1073. doi: 10.1785/0220130035.
